# Single-cell transcriptomics reveal that PD-1 mediates immune tolerance by regulating proliferation of regulatory T cells

**DOI:** 10.1186/s13073-018-0581-y

**Published:** 2018-09-20

**Authors:** Cherry S. Leung, Kevin Y. Yang, Xisheng Li, Vicken W. Chan, Manching Ku, Herman Waldmann, Shohei Hori, Jason C. H. Tsang, Yuk Ming Dennis Lo, Kathy O. Lui

**Affiliations:** 1 Department of Chemical Pathology, Prince of Wales Hospital, The Chinese University of Hong Kong, Hong Kong, China; 2 Department of Paediatrics and Adolescent Medicine, Division of Paediatric Hematology and Oncology, Faculty of Medicine, Medical Center, University of Freiburg, Freiburg im Breisgau, Germany; 3 Sir William Dunn School of Pathology, University of Oxford, Oxford, UK; 4 Laboratory of Immunology and Microbiology, Graduate School of Pharmaceutical Sciences, The University of Tokyo, Tokyo, Japan; 5 Li Ka Shing Institute of Health Sciences, Prince of Wales Hospital, The Chinese University of Hong Kong, Hong Kong, China

**Keywords:** Single-cell transcriptomics, Transplant tolerance, CD4^+^ regulatory T cells, PD-1, Human pancreatic beta cells

## Abstract

**Background:**

We have previously reported an antigen-specific protocol to induce transplant tolerance and linked suppression to human embryonic stem cell (hESC)-derived tissues in immunocompetent mice through coreceptor and costimulation blockade. However, the exact mechanisms of acquired immune tolerance in this model have remained unclear.

**Methods:**

We utilize the NOD.*Foxp3*^hCD2^ reporter mouse line and an ablative anti-hCD2 antibody to ask if CD4^+^FOXP3^+^ regulatory T cells (Treg) are required for coreceptor and costimulation blockade-induced immune tolerance. We also perform genome-wide single-cell RNA-sequencing to interrogate Treg during immune rejection and tolerance and to indicate possible mechanisms involved in sustaining Treg function.

**Results:**

We show that Treg are indispensable for tolerance induced by coreceptor and costimulation blockade as depletion of which with an anti-hCD2 antibody resulted in rejection of hESC-derived pancreatic islets in NOD.*Foxp3*^hCD2^ mice. Single-cell transcriptomic profiling of 12,964 intragraft CD4^+^ T cells derived from rejecting and tolerated grafts reveals that Treg are heterogeneous and functionally distinct in the two outcomes of transplant rejection and tolerance. Treg appear to mainly promote chemotactic and ubiquitin-dependent protein catabolism during transplant rejection while seeming to harness proliferative and immunosuppressive function during tolerance. We also demonstrate that this form of acquired transplant tolerance is associated with increased proliferation and PD-1 expression by Treg. Blocking PD-1 signaling with a neutralizing anti-PD-1 antibody leads to reduced Treg proliferation and graft rejection.

**Conclusions:**

Our results suggest that short-term coreceptor and costimulation blockade mediates immune tolerance to hESC-derived pancreatic islets by promoting Treg proliferation through engagement of PD-1. Our findings could give new insights into clinical development of hESC-derived pancreatic tissues, combined with immunotherapies that expand intragraft Treg, as a potentially sustainable alternative treatment for T1D.

**Electronic supplementary material:**

The online version of this article (10.1186/s13073-018-0581-y) contains supplementary material, which is available to authorized users.

## Background

The global prevalence of diabetes was 8.5% in 2014 [1] and is predicted to rise due to the growing obesity epidemic. Although most type 1 (T1D) and some type 2 (T2D) diabetic patients receive insulin therapy, it does not provide a real-time glycemic control to patients compared to transplantation of glucose-sensing, insulin-secreting pancreatic islets so patients are still at risk of developing hypoglycemia and cardiovascular complications [2]. Transplantation of human cadaveric pancreatic islets has been implemented clinically [3, 4] with a majority of patients achieving insulin independence within the first year but declining rapidly afterwards [3, 4]. Any therapeutic efficacy of islet transplantation has also been largely limited by the scarcity and quality of donor islets, chronic immune rejection [3], and recurrence of autoimmunity [5].

By virtue of their pluripotent and self-renewing properties, recent advances in human embryonic stem cell (hESC) technology have led to success in generating literally unlimited amount of human pancreatic endoderm cells or islets in vitro for transplantation, resulting in reversal of diabetes in mice [6–8]. Although transplantation of autologous induced pluripotent stem cell (iPSC) derivatives likely prevents immune rejection, T1D results from autoimmune attack so the T1D patient-specific iPSC-derived pancreatic beta cells might still harbor autoantigens to activate autoreactive memory T cells following transplantation [5]. On the other hand, direct presentation of autoantigens would be less likely with major histocompatibility complex (MHC) histoincompatible transplants. Moreover, cell therapy using derivatives of “off the shelf” hESCs is more economically and logistically feasible than hiPSCs when treating the population at large as well as patients with acute injuries [9]. Therefore, clinical trials using hESC derivatives [10, 11] and research in preventing their immune rejection still attract much attention [9, 12–14].

Transplantation of ESC-derived tissues also offers additional benefits [15–17] as they lack donor antigen-presenting cells (APCs) that elicit the direct pathway of allorecognition and are devoid of donor T cells that provoke graft-versus-host-disease (GVHD). All these features indicate that hESC-derived pancreatic tissues could be less immunogenic than human cadaveric islets for transplantation. We have previously reported that ESC-derived tissues exhibit some degree of immune privilege and can be spontaneously accepted by allogeneic recipients in conditions including disparity for a class-I MHC molecule [18].

Recently, clinical trials using encapsulated hESC-derived pancreatic endoderm cells have been approved in T1D patients [19, 20]. Cell encapsulation prevents direct contact between the transplanted cells and host immune system. Nevertheless, previous clinical studies have demonstrated that the therapeutic benefit of transplanting encapsulated human cadaveric islets into T1D patients is only temporary [21, 22]. This is likely due to the host’s innate immune response reacting to the implanted capsules, resulting in fibrosis, nutrient isolation, and donor tissue necrosis [23, 24]. To ensure long-term survival of the transplanted hESC-derived pancreatic tissues, particularly in autoimmune recipients with a primed immune system, the exploitation of endogenous tolerance processes will be invaluable.

One of the mechanisms by which antigen-specific immune tolerance established in mice operates through CD4^+^FOXP3^+^ regulatory T cells (Treg). Treg are indispensable for maintaining peripheral self-tolerance [25] and are important in suppressing allogeneic responses against non-self antigens during GVHD [26] or allograft rejection [18, 27]. Following allogeneic transplantation, Treg accumulating in tolerated grafts are immunosuppressive locally, can induce additional Treg (iTreg) from naïve T cells via infectious tolerance [27, 28], and can protect “third-party” antigens that coexist with tolerated antigens from immune rejection via linked suppression [29]. In all these situations, the mechanisms sustaining Treg survival in vivo are not yet fully understood.

Furthermore, it is unclear what Treg are doing within rejecting grafts. Are they solely bystanders, or remnants of a failed suppressive endeavor, or even contributors to the rejecting processes? We have previously reported an antigen-specific protocol to induce transplant tolerance and linked suppression to hESC-derived endothelial cells and neurons following transplantation into immunocompetent mice using coreceptor and costimulation antibody blockade [13]. Here, we demonstrate that such antibody blockade also promoted transplant tolerance to hESC-derived pancreatic islets in non-obese diabetic (NOD) mice that involved the activity of Treg. Using single-cell RNA-sequencing (scRNA-seq), we performed genome-wide characterization of 12,964 intragraft CD4^+^ T cells including conventional T cells and Treg of both rejecting and tolerated grafts. We show that conventional T cells found in the tolerated grafts expressed genes previously reported to support Treg function. Moreover, Treg were heterogeneous despite the beneficial transplant outcome. In contrast, there were at least two subsets of Treg in rejecting grafts that were distinct and less proliferative compared to those in tolerated grafts.

Our scRNA-seq data also show that antibody blockade augmented proliferation and PD-1 expression of Treg in tolerated grafts. Recently, immunotherapy targeting immune checkpoints including programmed cell death protein 1 (PD-1) has become a promising anti-cancer medicine. However, it has been reported that patients receiving anti-PD-1 antibodies developed T1D and other autoimmune diseases [30]. Although the role of PD-1 on conventional T cells has been well established [31], whether the PD-1 signaling can in some way affect Treg remains unclear. In this study, we observed that PD-1 blockade via a neutralizing anti-PD-1 antibody reduced proliferation of Treg and prevented tolerance induced by coreceptor and costimulation blockade. Taken together, our results suggest that coreceptor and costimulation blockade mediated transplant tolerance to hESC-derived pancreatic islets in NOD mice, at least in part, by promoting Treg proliferation via PD-1 signaling.

## Methods

### Human ESC cultures and pancreatic islet differentiation

The H9 hESC line (WA09, WiCell) was maintained in mTseR1 medium (Stemgent). EB induction was performed by resuspending hESCs in differentiation medium containing DMEM/F12, 10% knockout serum replacement (KOSR), 1X non-essential amino acids, 1X glutamine, 1X penicillin/streptomycin, and 1X b-mercaptoethanol (Gibco) in hanging drop cultures overnight. Individual EBs were then transferred to suspension cultures and incubated in differentiation medium containing 1% KOSR for another 12 days before transplantation. For pancreatic islet differentiation, hESCs were differentiated by stepwise administration of growth factors as described previously [6]. Briefly, a combination of growth factors was supplemented as follows: day 1/S1: 100 ng/ml Activin A (Peprotech) + 3 μM Chir99021 (Selleck Chem); day 2/S1: 100 ng/ml Activin A; days 4 and 6/S2: 50 ng/ml KGF (Peprotech); days 7 and 8/S3: 50 ng/ml KGF + 0.25 μM Sant1 (Sigma) + 2 μM RA (Sigma) + 200 nM LDN193189 (Sigma, on day 7 only) + 500 nM PdBU (EMD Millipore); days 9 and 11, 13/S3: 50 ng/ml KGF + 0.25 μM Sant1 + 100 nM RA; days 14 and 16/S5: 0.25 μM Sant1 + 100 nM RA + 1 μM XXI (Calbiochem) + 10 μM Alk5i II (Selleck Chem) + 1 μM T3 (EMD Millipore) + 20 ng/ml Betacellulin (Peprotech). Days 18 and 20/S5: 25 nM RA + 1 μM XXI + 10 μM Alk5i II + 1 μM T3 + 20 ng/ml Betacellulin. Days 21–35/S6 (medium changed on alternative days): 10 μM Alk5i II + 1 μM T3. In the final stage, cells were cultured in CMRL 1066 modified medium (CMRLM).

### Mice

*Foxp3*^hCD2^ reporter mice (C57BL/6) [32] were backcrossed onto the NOD/ShiJc1 (Clea Japan. Inc) background for 12 generations. Experiments were performed with mice at 8–10 weeks old before onset of diabetes.

### Kidney capsule transplantation

EBs or hESC-derived beta cell clusters were transplanted under the kidney capsule of NOD.Foxp3^hCD2^ mice as described previously [13, 18].

### Administration of monoclonal antibodies

Non-depleting mAb specific for CD4 (1 mg, clone YTS 177), CD8 (1 mg, clone YTS 105), and CD40L (1 mg, clone MR1, BioXcell) were injected intraperitoneally (i.p.) on days 0, 2, and 4 following transplantation. For Treg depletion, ablative anti-hCD2 mAb (0.25 mg, clone YTH655) was injected i.p. on days 0–7 following transplantation as previously described [27]. For PD1 blockade, neutralizing anti-PD1 mAb (0.5 mg, clone RMP1-14, BioXcell) was injected i.p. on days 0, 2, 4, 6, and 8 following transplantation as previously described [33]. The hybridoma lines for making YTS177, YTS105, and YTH655 antibodies were prepared as previously described [13, 18, 34].

### Immunostaining

Kidney grafts were dissected and fixed in 4% paraformaldehyde at 4 °C overnight. The fixed grafts were washed three times with PBS and equilibrated in 30% sucrose for 2 days before freezing and cryosectioning. Six-micrometer sections were blocked at 2% goat serum and then stained with the respective primary antibodies at 10 μg/ml at 4 °C overnight. Anti-human primary antibodies used are the following: PDX1 (R&D systems), NKX6.1 (R&D systems), GLUCAGON (Abcam), and C-PEPTIDE (DSHB), and the anti-mouse primary antibodies used are the following: Ki67 (eBiosciences) and FOXP3 (Cell Signaling Technology). Alexa-Fluor-488- or Alexa-Fluor-594-conjugated secondary antibodies (Invitrogen) were used at room temperature for 30 min in the dark. Slides were mounted with DAPI-containing fluorescence mounting medium (Dako), and fluorescence was detected with a confocal microscope (Leica). Some sections were also stained with hematoxylin and eosin (H&E) for histological analyses.

### FACS sorting and analysis

Splenocytes were dissociated by pressing the excised spleen in PBS with a syringe plunger through a 40-μm cell strainer to obtain single cell suspension. Single blood cells were dissociated from whole blood after removal of plasma in EDTA. Single graft cells were obtained by digesting the grafts with a digestion buffer containing collagenase II (11 U/ml, Worthington), dispase (1000 U/ml, Gibco), and DNase I (10 U) at 37 °C for 20–30 min. Enzymatic action was stopped by adding 10% FBS, and the dissociated cells were washed twice with PBS. The dissociated single splenocytes, blood, or graft cells were removed from the contaminated erythrocytes by incubating with the red blood cell lysis buffer (eBiosciences) for 5 min and were then blocked with 2% heat-inactivated rabbit serum. Cells were subsequently stained with fluorochrome-conjugated antibodies against the following antigens: mCD3, mCD4, mPD1, or hCD2 (Biolegend) at a dilution of 1:100, unless specified by the manufacturer, at 4 °C for 30 min. Murine Treg were detected with the Treg staining kit according to the manufacturer’s instructions (eBioscience). Cells were then washed three times with 2% FBS-containing PBS and analyzed on flow cytometer (BD FACSAria™ Fusion). Propidium iodide (PI, BD) positive dead cells were excluded for live cell analysis/sorting, and FACS data were then analyzed with the FlowJo software (Tree star).

### Bulk RNA-sequencing and functional annotations

Total RNA was isolated from FACS-sorted cells using the RNeasy mini kit (Qiagen) and analyzed on the Agilent Tape station for RNA Integrity Numbers (RIN) prior to library preparation. RNA-Seq libraries were prepared using TruSeq Stranded mRNA Library Prep Kit according to manufacturer’s protocol (Illumina). mRNA was isolated using poly-T oligos conjugated to magnetic beads and then fragmented and reverse-transcribed to cDNA. dUTPs were incorporated during second-strand synthesis and thus not amplified. cDNA was then undergone end-repair, ligation with indexed adapters, and PCR amplification. Nucleic acid was cleaned up after each steps using AMPure XP beads (Beckman Coulter). Libraries were then quantified, pooled, and sequenced at single-end 50 base-pair on the Illumina HiSeq platform. Libraries were sequenced at an average depth of 20 million reads per library. After trimming low-quality bases, the sequenced reads were aligned to the mouse reference genome (mm10) using STAR (v2.4.2a) with default settings [35]. Reference genome and gene model file (mm10) were obtained from HOMER [36]. The expression abundances of all genes and differentially expressed genes were calculated by HOMER (v4.7) with default parameters. The identified differentially expressed genes were further annotated with Gene Ontology (GO) using DAVID Bioinformatics Resources (v6.8) [37].

### Single-cell encapsulation and library preparation

Single cells were purified by FACS sorting before library preparation, and single-cell libraries were prepared with the Chromium Single Cell 3’ Reagent Kits v2 (10x Genomics) as per manufacturer’s instructions. Briefly, sorted cells in suspension were first prepared as gel beads in emulsion (GEMs) on Single Cell 3’ Chips v2 (10x Chromium) using the Chromium Controller (10x Genomics). Barcoded RNA transcripts in each single cell were reverse transcribed within GEM droplets. cDNA was purified with DynaBeads MyOne Silane beads (Invitrogen) and then amplified for subsequent library construction. Sequencing libraries were prepared by fragmentation, end-repair, ligation with indexed adapters, and PCR amplification using the Chromium Single Cell 3’ library kit v2 (10x Genomics). Nucleic acid was cleaned up after each steps using SPRIselect beads (Beckman Coulter). Libraries were then quantified by Qubit and real-time quantitative PCR on a LightCycler 96 System (Roche).

### Single-cell RNA-sequencing and functional annotations

Pooled libraries were sequenced on the Illumina NextSeq 500 platform. All single-cell libraries were sequenced with a customized paired-end dual index format (98/26/0/8 basepair) according to manufacturer’s instructions. Data were processed, aligned, and quantified using the Cell Ranger Single-Cell Software Suite (v 2.0) [38]. Briefly, data were demultiplexed based on the 8 base-pair sample index, 16 base-pair Chromium barcodes, and 10 base-pair unique molecular identifiers (UMI). Two distinct groups of contaminated cells were removed as they expressed genes of the myeloid lineage. To eliminate the impact of cell number bias, data from ~ 1000 cells of each sample were randomly selected for further analysis. Cells with either very low or too high mRNA content (i.e., out of two standard deviations) or high fractions of mitochondrial encoded transcripts (> 10%) were filtered out. Data were aligned on *Mus musculus* Cell Ranger transcriptome reference (mm10-1.2.0), and analyses, including PCA, tSNE, and graph-based clustering, were performed according to Cell Ranger’s pipelines with default settings. To perform differential expression analysis on each comparison, Cell Ranger’s pipelines were applied with sSeq algorithm [39], which employs a negative binomial exact test to generate *p* values and further adjusted using Benjamini-Hochberg. To perform GO functional enrichment analysis, genes that satisfy a less stringent criterion (with at least fourfold changes) were considered to be potential targets, which were further annotated with GO using DAVID Bioinformatics Resources (v6.8) [37]. Cell cycle phase classifications were performed by scran [40] with default settings.

### Statistical analysis

The data were expressed as arithmetic mean ± s.d. of biological replicates (*n* = 6, unless otherwise specified) performed under the same conditions. Statistical analysis was performed using the unpaired Student’s *t* test with data from two groups, while data from more than two groups was performed using an ANOVA followed by Tukey’s method for multiple comparisons. Significance was accepted when *P* < 0.05.

## Results

### Coreceptor and costimulation blockade facilitates survival and maturation of hESC-islets in NOD mice

**Fig. 1:**
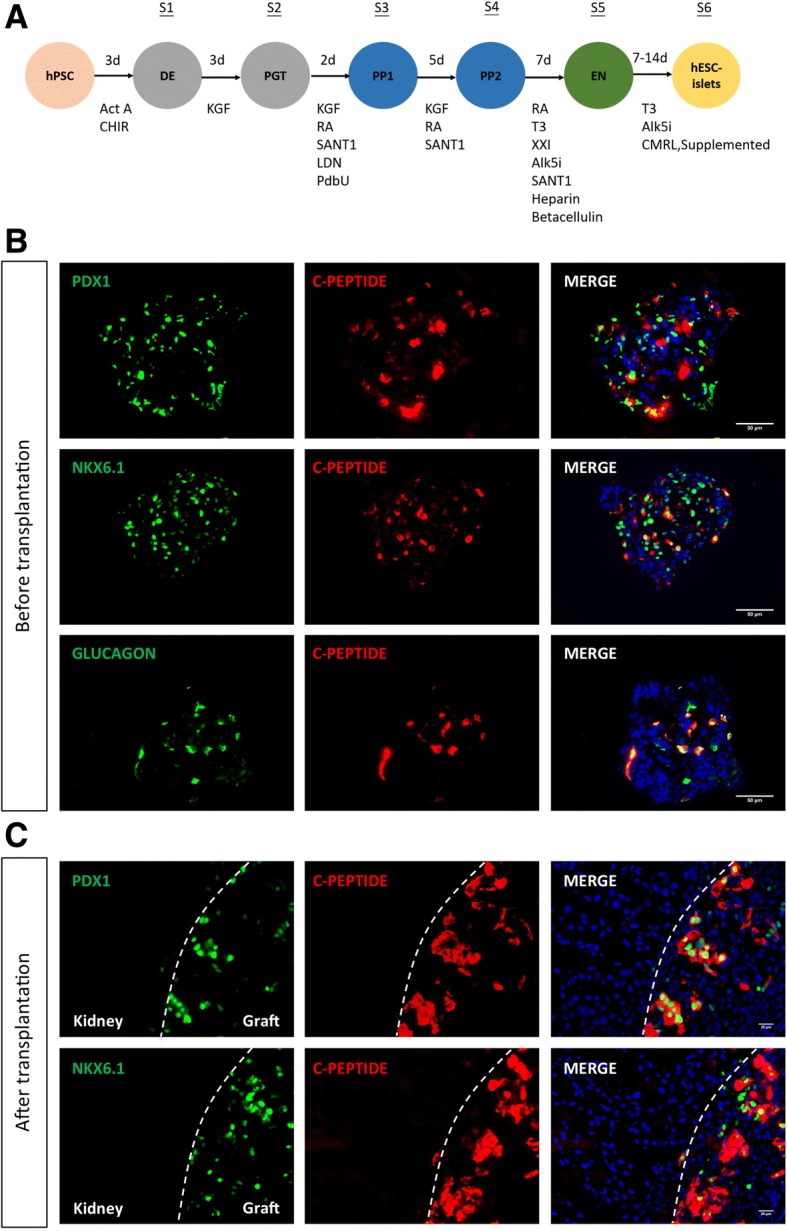
Coreceptor and costimulation blockade facilitates survival and maturation of hESC-derived pancreatic islets in NOD.Foxp3^hCD2^ mice. **a** A schematic diagram showing the simplified stepwise differentiation protocol to generate human pancreatic islets from hESCs. **b**, **c** Immunostaining for the lineage-specific markers of human pancreatic islets **b** before and **c** after 1-month transplantation of hESC-islets in NOD.*Foxp3*^hCD2^ mice (*n* = 6) following treatment with coreceptor and costimulation blockade. Scale bars in **b**: 50 μm and in **c**: 20 μm

We have previously reported that coreceptor and costimulation blockade induces transplant tolerance and linked suppression to hESC-derived progenitor cells and their differentiated progenies in immunocompetent mice [13]. To ask if the same regimen protects grafts from rejection in mice with an autoimmune background such as NOD, we transplanted surrogate hESC-derived embryoid bodies (hESC-EB) under the kidney capsule of 10-week-old NOD mice under cover of treatment with anti-CD4, anti-CD8, and anti-CD40L monoclonal antibodies (3 mAb), previously shown to allow transplants to survive for at least 3 months as previously described [13]. We found that hESC-EB survived and differentiated into three embryonic germ layers at 1 month after transplantation (Additional file 1: Figure S1), indicating that coreceptor and costimulation blockade induced graft acceptance in NOD mice. In fact, coreceptor blockade alone can reverse hyperglycemia in NOD mice as previously described [41]. Moreover, we differentiated pancreatic islets from hESCs (hESC-islets) as a surrogate tissue for transplantation (Fig. 1a) using a protocol previously demonstrated to reverse streptozotocin-induced hyperglycemia [6]. Whether the surrogate tissue reversed hyperglycemia was not the focus of this study as both the tolerance induction regimen and surrogate tissues facilitate remission of diabetes. Rather, we studied the mechanisms by which coreceptor and costimulation blockade induced transplant tolerance to hESC-derived tissues. This is why we transplanted 10-week-old NOD mice, well before the onset of diabetes, to minimize the risk that autoimmune reactions might alter analysis of mechanisms operated during induction of transplant tolerance.

Immunostaining for human PDX1, NKX6.1, or GLUCAGON with C-PEPTIDE, respectively, in stage 6 cells confirmed differentiation of hESCs into hESC-islets (Fig. 1b). We then transplanted hESC-islets under cover of treatment with 3 mAb and found that hESC-islets survived in NOD mice without teratoma formation (Fig. 1c). Moreover, immunostaining for human PDX1 or NKX6.1 with C-PEPTIDE in tolerated grafts revealed that hESC-islets matured in vivo as there were more PDX1^+^C-PEPTIDE^+^ or NKX6.1^+^C-PEPTIDE^+^ cells in tolerated grafts (Fig. 1c) than those before transplantation (Fig. 1b). Our results demonstrated that hESC-islets served as a surrogate tissue for downstream analysis, and coreceptor and costimulation blockade induced transplant tolerance to hESC-derived tissues not only in wildtype but also in recipients of T1D background not yet exhibiting autoimmune disease.

### Coreceptor and costimulation blockade promotes transplant tolerance to hESC-derived tissues through CD4^+^ Treg

**Fig. 2:**
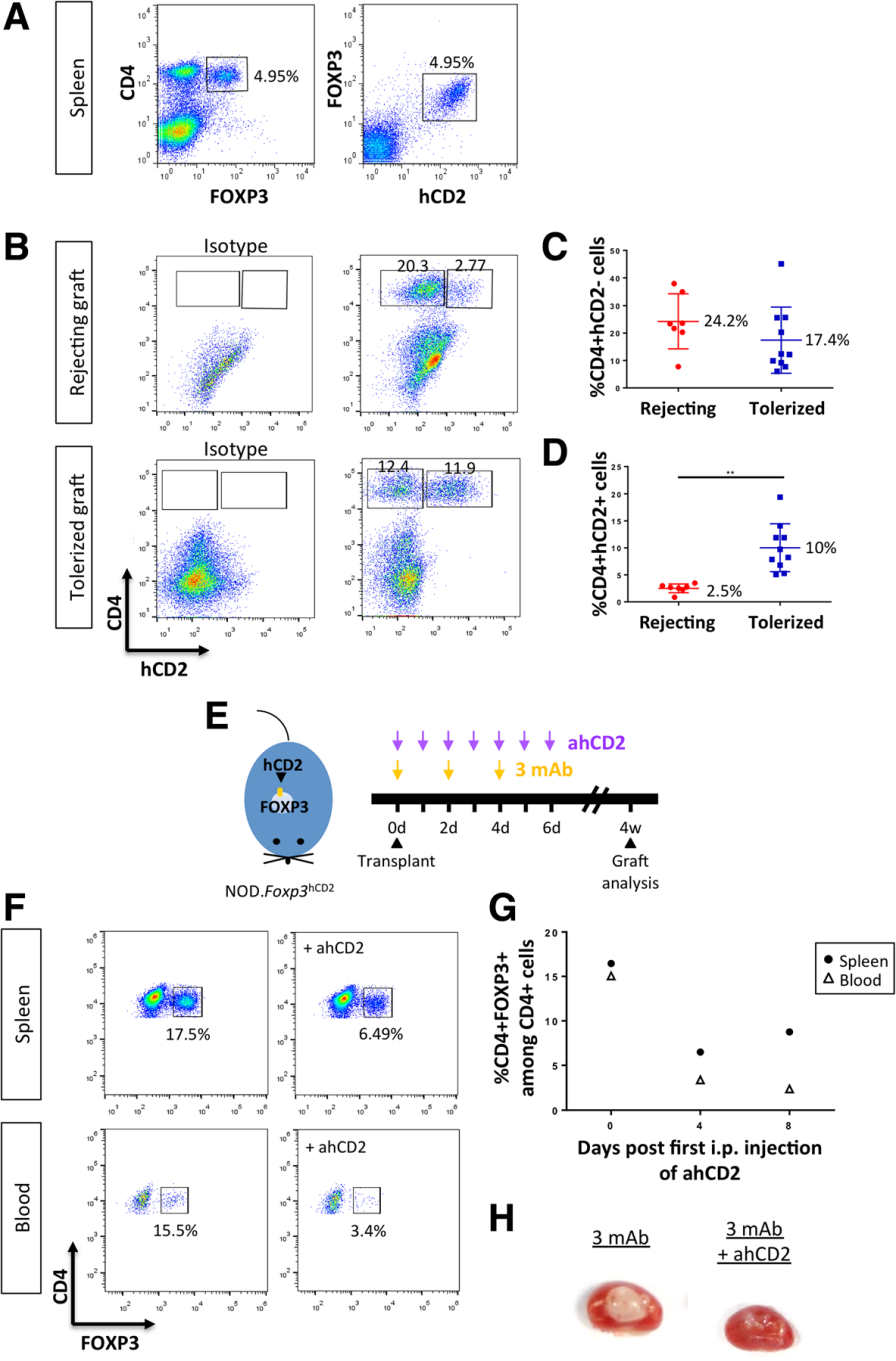
Coreceptor and costimulation blockade promotes transplant tolerance to hESC-derived tissues through CD4^+^ Treg. **a** Flow cytometric analysis showing surface expression of hCD2 by all CD4^+^FOXP3^+^ Treg in NOD.Foxp3^hCD2^ mice. **b** Flow cytometric analysis and **c**, **d** quantifications showing **c** comparable infiltration of CD4^+^hCD2^−^ conventional T cells but **d** significantly increased infiltration of CD4^+^hCD2^+^ Treg in tolerated (*n* = 10) than rejecting (*n* = 10) grafts at 1 month post-transplantation. **P < 0.01. **e** A schematic diagram showing the protocol for antibody treatments. **f** Flow cytometric analysis and **g** time-dependent quantifications showing reduced percentage of CD4^+^FOXP3^+^ Treg among total CD4^+^ T cells in the spleen and blood of NOD.*Foxp3*^hCD2^ mice after treatment with the ablative αhCD2 antibody. **h** Representative images showing that αhCD2 antibody abolished coreceptor and costimulation blockade-mediated tolerance to hESC-islets at 1 month following transplantation, *n* = 6 per group

Although Treg deficiency or dysfunction is sufficient to break self-tolerance [25], it remains unclear whether they are indispensable for maintaining transplant tolerance to hESC-derived tissues. Previously, we were unable to detect CD4^+^FOXP3^+^ Treg in rejecting grafts and very few were detected in tolerated grafts [13] due to cell loss through intra-nuclear immunostaining for FOXP3 and to a lack of reliable cell surface marker(s) for phenotyping of murine CD4^+^ Treg. To examine contribution of Treg in coreceptor and costimulation blockade-mediated transplant tolerance to hESC-derived tissues, we backcrossed the *Foxp3*^hCD2^ reporter “knockin” allele [32] onto the NOD background, which allowed us to purify Treg via their surface expression of hCD2. We first confirmed co-localization of FOXP3 and hCD2 in NOD.*Foxp3*^hCD2^ mice by flow cytometry (Fig. 2ba). We also detected CD4^+^hCD2^+^ Treg in both rejecting and tolerated hESC-derived grafts (Fig. 2), while there was no significant difference in percentage of CD4^+^hCD2^−^ conventional T cells (Th) of rejecting and tolerated grafts (Fig. 2c); a significantly higher percentage of CD4^+^hCD2^+^ Treg was found in tolerated than rejecting grafts (Fig. 2d).

To determine if Treg were necessary for antibody-mediated tolerance, we depleted them with an ablative anti-hCD2 mAb (αhCD2, Fig. 2e). On day 4 post-αhCD2 treatment, we already observed a threefold and fivefold reduction in splenic and circulatory CD4^+^FOXP3^+^ Treg within the CD4^+^ population, respectively (Fig. 2f). In fact, the depletion efficiency of Treg by αhCD2 in NOD.*Foxp3*^hCD2^ (Fig. 2g) was comparable to that in B6.*Foxp3*^hCD2^ as previously described [27]. We then transplanted hESC-islets in NOD.*Foxp3*^hCD2^ and examined graft survival at 1 month following transplantation with 3 mAb or 3 mAb + αhCD2 mAb. Compared to grafts derived from 3 mAb-treated group that were 100% accepted, those from 3 mAb + αhCD2 mAb-treated group were 100% rejected (*n* = 6 per group, Fig. 2h). Our results indicated that Treg were indispensable for coreceptor and costimulation blockade-induced transplant tolerance.

### Genome-wide transcriptomic profiling of splenic CD4^+^ Treg during transplant rejection and tolerance

We next asked if Treg were beginning to influence transplant outcome in secondary lymphoid tissues by purifying splenic CD4^+^hCD2^+^ Treg from 3 mAb + αhCD2 mAb-treated rejecting and 3 mAb-treated tolerized NOD.*Foxp3*^hCD2^ following transplantation for bulk RNA-seq. We found 43 differentially expressed genes (Additional file 1: Figure S2), and functional annotations by GO showed that the most significantly downregulated genes in splenic Treg of tolerized compared to rejecting mice were associated with neutrophil chemotaxis (*Csf3r*, *Ccl1*, *Itga1*, *Il1b*, *Ccl5*), angiogenesis (*Mmp9*, *Il1b*, *Lrg1*, *Ccl5*), and regulation of T cell proliferation (*Il21*, *Il1b*, *Ccl5*). Even though a total of 24,020 genes were identified in splenic Treg, this paucity of differentially expressed genes might indicate that Treg were largely similar in secondary lymphoid tissues during transplant rejection and tolerance.

### Genome-wide transcriptomic profiling of intragraft CD4^+^ Th and Treg during transplant rejection and tolerance at single-cell resolution

Previous reports have suggested that Treg-mediated tolerance to allogeneic skin grafts mainly operates at the graft site [27, 42]; however, the identities and interrelationships of different CD4^+^ T cell subsets at the graft site during transplant rejection and tolerance have not been studied. The definition of different CD4^+^ T cell subsets is likely biased due to insufficient surface markers for their purification and manipulation. To overcome this, we sought to understand the heterogeneity of CD4^+^ T cells through large-scale droplet-based single-cell transcriptomic profiling [38, 43]. In this system, individual cells were encapsulated in microfluidic droplets with unique nucleotide barcodes and molecule identifiers (UMI) for tagging RNAs inside the droplets. We purified about ~ 12,964 intragraft CD4^+^ T cells by FACS including 858 CD4^+^hCD2^−^ helper T cells (Th) and 954 CD4^+^hCD2^+^ Treg from ten recipients with rejecting grafts (untreated controls) and 3654 Th and 7498 Treg from ten recipients with tolerated grafts (3 mAb treated). It is worthy of note that untreated mice were used as rejecting controls as Treg depletion via anti-hCD2 antibodies could change the T cell populations within the grafts that could interfere our heterogeneity analysis. Moreover, to eliminate the impact of cell number bias, data from ~ 1000 cells of each sample were randomly selected for further analysis (Additional file 1: Figure S3).

**Fig. 3:**
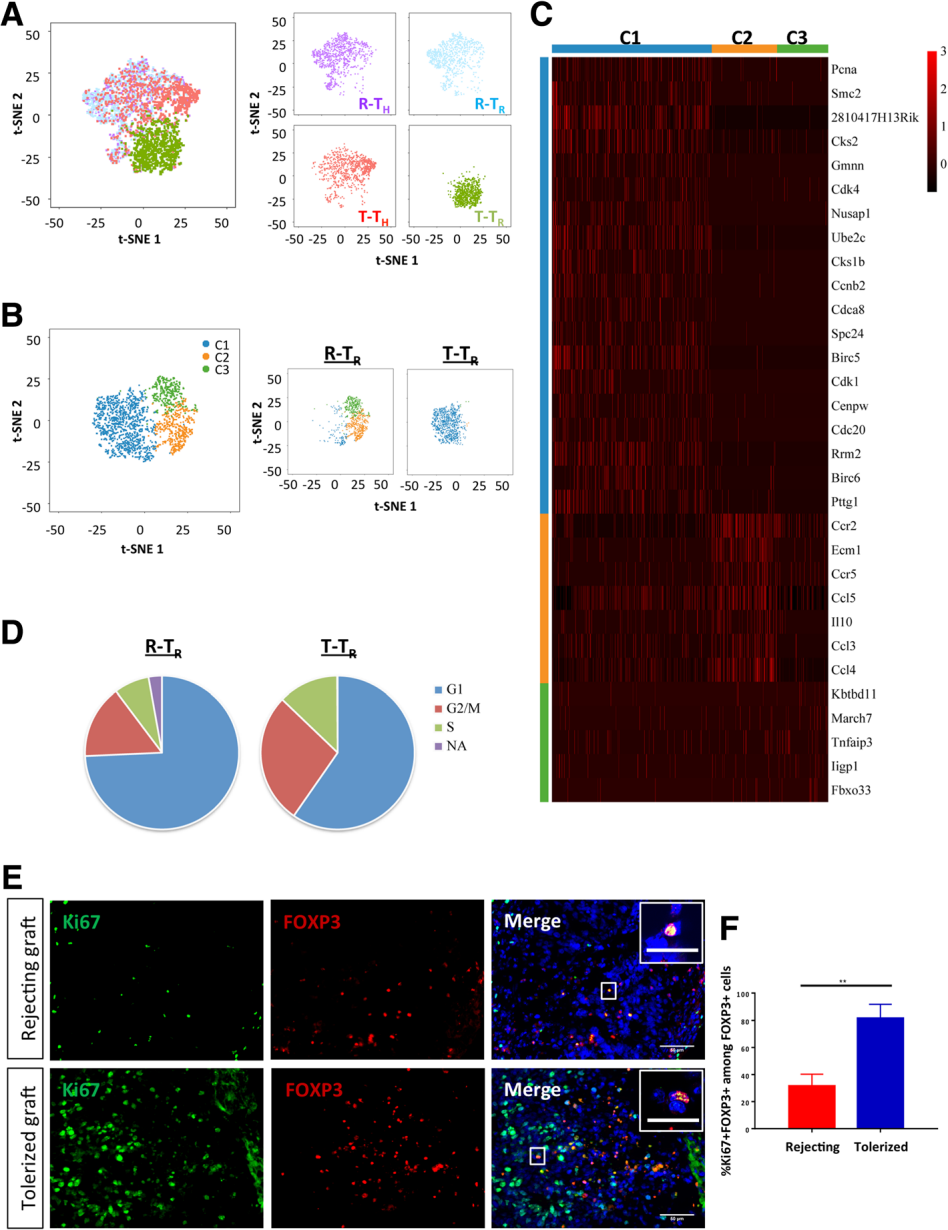
Coreceptor and costimulation blockade-induced transplant tolerance is predominantly mediated by intragraft proliferation of CD4^+^ Treg. **a** Biaxial scatter plots by *t*-SNE analysis showing single-cell transcriptomic clustering of ~ 1000 CD4^+^hCD2^−^ (T_H_) or ~ 1000 CD4^+^hCD2^+^ (T_R_) cells purified from rejecting and tolerated grafts, respectively. Cells were colored individually according to their initial expression of CD4 and hCD2 during FACS sorting. **b** *t*-SNE analysis showing single-cell transcriptomic clustering of ~ 1000 CD4^+^hCD2^+^ Treg cells during transplant rejection and tolerance, respectively. Cells were further subgrouped into specific clusters (C1-4) and colored individually according to expression patterns of specific marker genes and spatial proximity in the biaxial plot. **c** From selected pathways determined by GO functional annotations in terms of biological processes of each cluster (Additional file 1: Tables S4–S6), upregulated genes with an average expression level > 0.05 were displayed by the heatmap. **d** Scran analysis showing cell cycle phase classifications. **e** Immunostaining and **f** quantifications of Ki67^+^FOXP3^+^ cells among total FOXP3^+^ cells in rejecting and tolerated grafts. Inserts denote magnified Ki67^+^FOXP3^+^ cells. Scale bars in **e**: 50 μm and inserts: 10 μm. *n* = 6 per group, **P < 0.01. Abbreviations: R-T_H_, CD4^+^hCD2^−^ Th of rejecting grafts; R-T_R_, CD4^+^hCD2^+^ Treg of rejecting grafts; T-T_H_, CD4^+^hCD2^−^ Th of tolerated grafts; and T-T_R_, CD4^+^hCD2^+^ Treg of tolerated grafts

We defined four cell clusters according to their initial CD4 and hCD2 expression and performed unsupervised analysis that did not rely on other known CD4^+^ T cell subset markers. From the *t*-distributed stochastic neighbor embedding (*t*-SNE) plots, we observed that CD4^+^hCD2^−^ Th (R-T_H_) and CD4^+^hCD2^+^ Treg (R-T_R_) of rejecting grafts mapped closely together; CD4^+^hCD2^−^ Th of tolerated grafts (T-T_H_) mapped closer to R-T_H_ and R-T_R_ than CD4^+^hCD2^+^ Treg of tolerated grafts (T-T_R_); and T-T_R_ formed a distinct population (Fig. 3a). Based on their cytokine expression profiles, we further characterized CD4^+^ Th subsets during transplant rejection and tolerance. R-T_H_ included *Ifng*-expressing Th1 cells, *Il4*, *Il5*, and *Il13*-expressing Th2 cells as well as Il21-expressing Th17 cells during rejection, and T-T_H_ mainly included *Ifng*-expressing Th1 cells as well as *Il21*-expressing Th17 cells during tolerance (Additional file 1: Figure S4A).

Next, we performed pairwise analysis to identify the differentially expressed genes. Although R-T_H_ and T-T_H_ did not form distinct clusters on *t*-SNE (Additional file 1: Figure S5A), 392 differentially expressed genes were identified in T-T_H_ compared to R-T_H_ and GO functional annotations showed that the most significantly upregulated pathway was associated with negative regulation of the immune system such as expression of *Cd81* [44] and *Ccr7* [45] that support Treg function or *Tmem176a* and *Tmem176b* that negatively regulate dendritic cell differentiation. Moreover, the most significantly downregulated pathways were associated with responses to interferon-α/β/γ (Additional file 1: Figure S5B, gene listed in Additional file 1: Table S1). Therefore, CD4^+^ Th cells might, perhaps, elicit more immunomodulatory than inflammatory responses during transplant tolerance than rejection.

During transplant rejection, we found that R-T_R_ and R-T_H_ mapped closely together on *t*-SNE (Additional file 1: Figure S5C). A total of 367 differentially expressed genes were identified in R-T_R_ compared to R-T_H_, and GO functional annotations showed that the most significantly upregulated pathways were associated with negative regulation of conventional T cell function (e.g., *Cd81*, *Phlpp1*, *Foxp3*, *Sdc4*, and *Il2ra*) and the most significantly downregulated pathways were associated with adaptive immune responses (e.g., *Cd40Ig*, *Il2*, *Il4*, *Ifng*), DNA repair, and autophagy (Additional file 1: Figure S5D, gene listed in Additional file 1: Table S2). Compared to CD4^+^ Th cells, Treg during transplant rejection could still harbor anti-inflammatory functions.

During transplant tolerance, we found that T-T_R_ and T-T_H_ formed distinct clusters on *t*-SNE (Additional file 1: Figure S5E). A total of 565 differentially expressed genes were identified in T-T_R_ compared to T-T_H_, and GO functional annotations showed that the most significantly upregulated pathways were associated with cell proliferation and antigen presentation via MHC-II (e.g., *H2-Aa*, *Cd74*, *H2-Ab1*, *Ifi30*, *H2-Eb1*) and the most significantly downregulated pathways were associated with regulation of B cell/T cell proliferation (e.g., *Cd40Ig*, *Nckap1l*, *Tnfsf13b*, *Tnfrsf13c*, *Bcl2*, *Bmi1*, *Ccl5*, *Cd274*) and NK cell chemotaxis (e.g., *Ccl3*, *Ccl5*, *Xcl1*, Additional file 1: Figure S5F, gene listed in Additional file 1: Table S3). Compared to CD4^+^ Th cells, Treg during transplant tolerance could replicate with immunosuppressive functions.

### Intragraft CD4^+^ Treg are phenotypically and functionally distinct during transplant rejection and tolerance

To further examine whether Treg are bystanders during transplant rejection, we identified their specific phenotypic and functional differences by comparing their transcriptomic signatures during transplant tolerance. In addition to FOXP3/hCD2, R-T_R_ and T-T_R_ also expressed other Treg markers including *Il2ra*, *Ikzf2*, *Nrp1*, and *Il10* (Additional file 1: Figure S4B). Nevertheless, they formed distinct clusters on *t*-SNE: all T-T_R_ but few R-T_R_ formed cluster C1, and a majority of R-T_R_ formed clusters C2 and C3 (Fig. 3b, Table 1). Comparing differentially expressed genes of these clusters by GO functional annotations, C1 was distinguished from the other clusters by upregulated cell cycle/cell division genes and downregulated inflammatory/chemotactic genes as demonstrated by heat map (Fig. 3c) and pathway analyses (Additional file 1: Figure S6A, gene listed in Additional file 1: Table S4). Similarly, C2 was marked by upregulated chemotactic/chemokine genes and downregulated cell cycle/cell division genes (Additional file 1: Figure S6B, gene listed in Additional file 1: Table S5); C3 was characterized by upregulated genes regulating protein ubiquitination and responses to interferons and downregulated DNA/cell proliferation genes (Additional file 1: Figure S6C, gene listed in Additional file 1: Table S6). Altogether, with unbiased transcriptomic classification of FOXP3-expressing R-T_R_ and T-T_R_, we discovered that Treg could be more heterogeneous during transplant rejection than tolerance and they skewed away from replicating, immunosuppressive C1 to less replicating, chemotactic C2 or ubiquitination-prone C3.

**Table 1:** Distribution of cell number and percentage of CD4^+^ Treg during rejection and tolerance in each cell cluster as determined by *t*-SNE

Cluster	Total cell number (#)	# R-T_R_	# T-T_R_	% R-T_R_	% T-T_R_
1	1086	134	952	14.6	99.27
2	444	441	3	48.04	0.31
3	347	343	4	37.36	0.42

*R-T*_*R*,_ CD4^+^hCD2^+^ Treg of rejecting hESC-derived grafts; *T-T*_*R*,_ CD4^+^hCD2^+^ Treg Treg of tolerated hESC-derived grafts

### Coreceptor and costimulation blockade-induced transplant tolerance is associated with intragraft proliferation of CD4^+^ Treg

We also performed cell cycle phase classification analyses with the scRNA-seq data of R- T_R_ and T-T_R_ by Scran (Fig. 3d, Table 2). Our results showed that a larger proportion of T-T_R_ (~ 40%) than R-T_R_ (~ 22%) was found in the S-G2/M phases, implying DNA/cell proliferation. Moreover, we also validated our transcriptomic results by immunostaining for the proliferation marker Ki67 (Fig. 3e). Indeed, our results revealed that significantly more Ki67^+^FOXP3^+^ Treg could be found in tolerated than rejecting grafts (Fig. 3f). Therefore, it is likely that Treg proliferation was a significant mechanism by which coreceptor and costimulation blockade induced transplant tolerance to hESC-derived tissues.

**Table 2:** Distribution of cell number and percentage of CD4^+^ Treg during rejection and tolerance in each of the cell cycle phases as determined by Scran

	# R-T_R_	# T-T_R_	% R-T_R_	% T-T_R_
Total cell number (#)	918	959		
G1	682	572	74.29	59.65
G2M	142	264	15.47	27.53
S	68	122	7.41	12.72
NA	26	1	2.83	0.1

*R-T*_*R*_, CD4^+^hCD2^+^ Treg of rejecting hESC-derived grafts; *T-T*_*R*_, CD4^+^hCD2^+^ Treg Treg of tolerated hESC-derived grafts

### Proliferation of CD4^+^ Treg in tolerated grafts requires functional PD-1 signaling

**Fig. 4:**
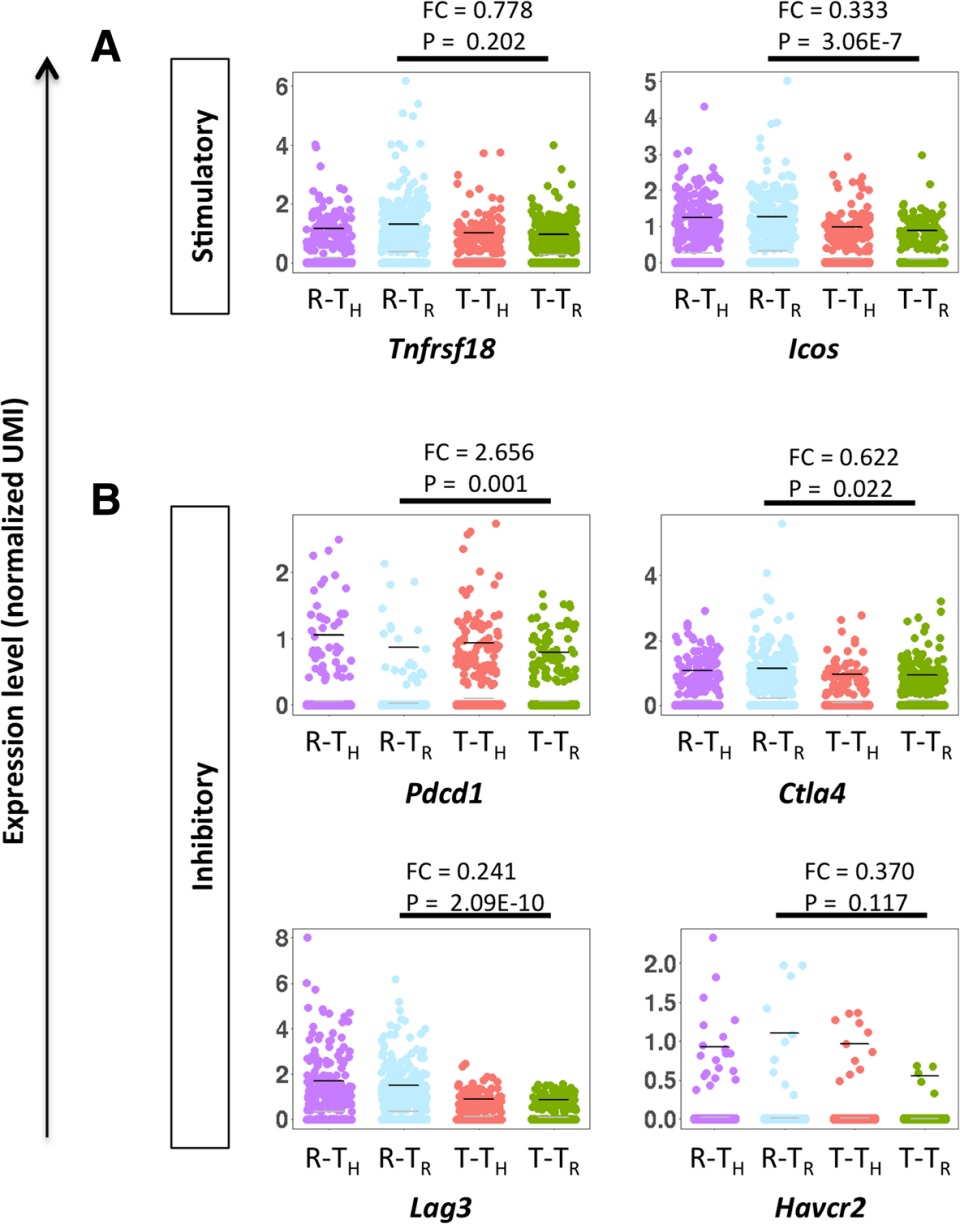
Identification of immune checkpoint-specific genes expressed by CD4^+^ T cells in rejecting and tolerated grafts. Jitter plots comparing expression levels of **a** stimulatory and **b** inhibitory immune checkpoint-specific genes expressed by CD4^+^hCD2^−^ Th of rejecting grafts (R-T_H_), CD4^+^hCD2^+^ Treg of rejecting grafts (R-T_R_), CD4^+^hCD2^−^ Th of tolerated grafts (T-T_H_), and CD4^+^hCD2^+^ Treg of tolerated grafts (T-T_R_). The fold change (FC) of T-T_R_ over R-T_R_ and the *p* value (P) by sSeq method are provided. Gray and black bars indicate the average expression level among all and expressed cells, respectively

**Fig. 5:**
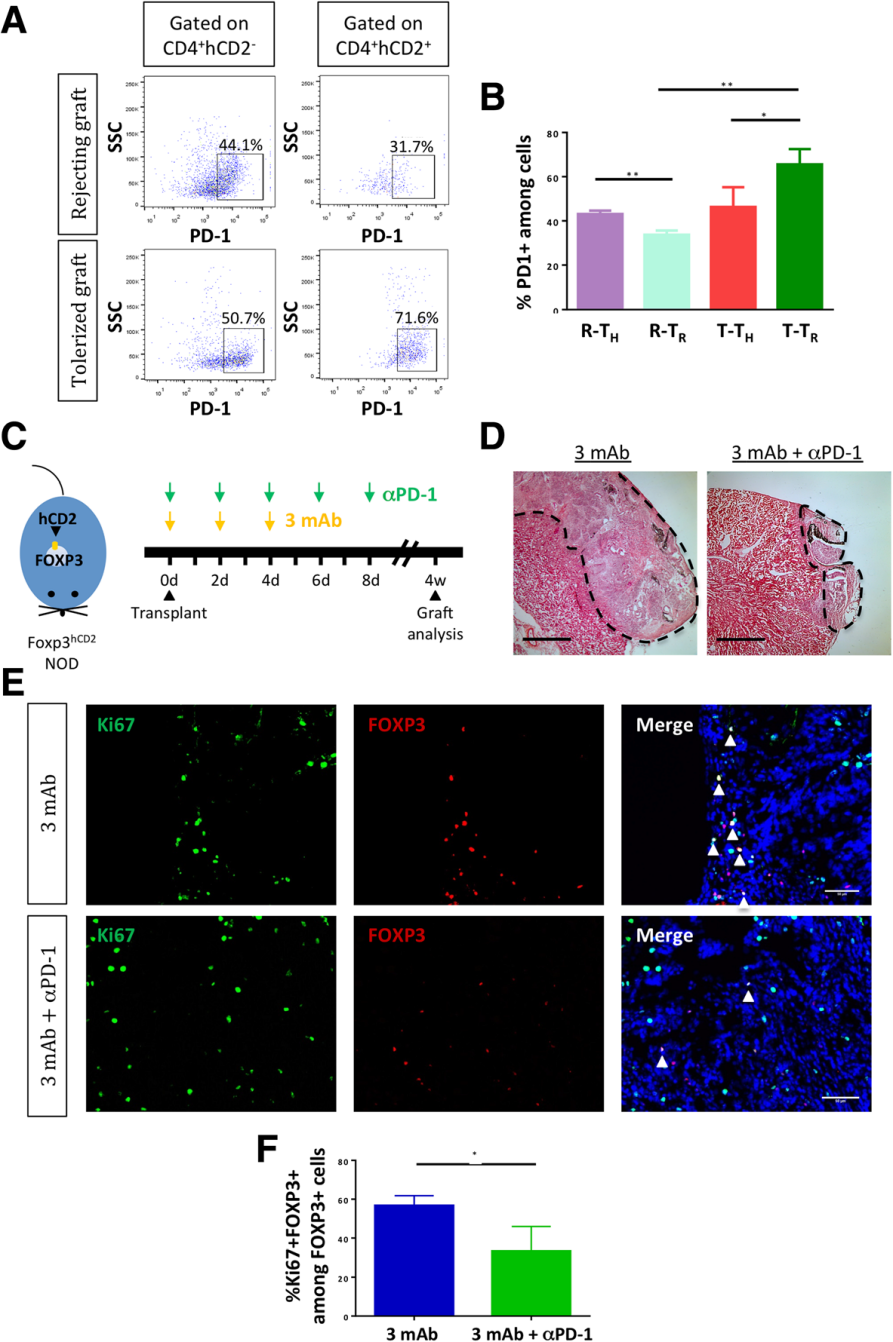
Proliferation of CD4^+^ Treg in tolerated grafts requires functional PD-1 signaling. **a** Flow cytometric analysis and **b** quantification showing expression of PD-1 in CD4^+^hCD2^−^ (T_H_) or CD4^+^hCD2^+^ (T_R_) cells of rejecting and tolerated grafts, respectively. **c** A schematic diagram showing the protocol for antibody treatments. **d** H&E staining showing graft rejection following treatment with αPD-1 mAb in addition to coreceptor and costimulation blockade (3 mAb). Scale bars: 1000 μm. **e** Immunostaining and **f** quantifications of Ki67^+^FOXP3^+^ cells among total FOXP3^+^ cells in 3 mAb- and 3 mAb + αPD-1 mAb-treated grafts, respectively. Arrows indicate Ki67^+^FOXP3^+^ cells. Scale bars: 50 μm. *P < 0.05. (**a**–**f**) *n* = 5 per group

We then examined the mechanisms by which coreceptor and costimulation blockade induced tolerance through promoting intragraft Treg proliferation. From our transcriptomic profiling data, we observed that expression of *Pdcd1*/PD-1 significantly increased while expression of other stimulatory (*Tnfrsf18/*GITR, *Icos*) and inhibitory (*Ctla4*, *Lag3*, *Havcr2*/TIM-3) checkpoint molecules remained relatively stable or significantly reduced in CD4^+^ T cells during transplant tolerance compared to rejection (Fig. 4). We, therefore, focused on PD-1 for functional validation. We confirmed our scRNA-seq results by flow cytometry that a significantly greater percentage of PD-1^+^CD4^+^hCD2^+^ population was found in tolerated than rejecting grafts, suggesting that coreceptor and costimulation blockade increased PD-1 expression by Treg during tolerance (Fig. 5a, b). Moreover, it appears that there was significantly higher PD-1 expression by CD4^+^hCD2^−^ than CD4^+^hCD2^+^ cells during rejection and vice versa during tolerance (Fig. 5b), implying that PD-1 was more predominantly expressed by the subset responsible for determining transplant outcome, i.e., more PD-1 expressed by Th during rejection and by Treg during tolerance.

With advances in immunotherapy using αPD-1 mAb in cancer patients, the inhibitory role of PD-1 signaling on conventional T cell activation has been well characterized [31]. However, whether PD-1 signaling regulates Treg proliferation and function, especially in the transplantation setting, remains elusive. To address this, we transplanted hESC-derived tissues in NOD.*Foxp3*^hCD2^ and examined graft survival in the 3 mAb- or 3 mAb + αPD-1 mAb-treated group at 1 month following transplantation (Fig. 5c). Compared to grafts derived from 3 mAb-treated group that were 100% accepted, blocking PD-1 signaling by αPD-1 mAb resulted in graft rejection (*n* = 5 per group, Fig. 5d). To investigate if PD-1 signaling regulated coreceptor and costimulation blockade-induced Treg proliferation, we performed immunostaining for Ki67 and FOXP3 in grafts derived from recipients treated with 3 mAb or 3 mAb + αPD-1 mAb (Fig. 5e). Our results revealed that significantly less Ki67^+^FOXP3^+^ Treg were found in the 3 mAb + αPD-1 mAb- than 3 mAb-treated group (Fig. 5f). Therefore, our results suggested that blocking PD-1 signaling negated the effect of coreceptor and costimulation blockade on Treg proliferation for maintaining transplant tolerance.

## Discussion

Despite the large body of evidence showing that CD4^+^FOXP3^+^ Treg play an important role in maintaining transplant tolerance, clinical studies demonstrate a correlation between intragraft CD4^+^FOXP3^+^ cells [46] or urinary *FOXP3* mRNA [47] and acute renal allograft rejection. Nevertheless, whether Treg mediated transplant tolerance is a numbers game or whether they are just “failed” bystanders during transplant rejection remains unknown. Since Treg determine the outcome of both autoimmunity and transplant rejection, we transplanted surrogate tissues in NOD recipients without ongoing autoimmunity in this study. We showed that Treg were indispensable for enabling coreceptor and costimulation blockade-mediated transplant tolerance to hESC-islets in NOD.*Foxp3*^hCD2^ recipients as their depletion with an ablative anti-hCD2 antibody led to graft rejection. We also found significantly more Treg resided in tolerated than rejecting grafts and showed that they were phenotypically distinct at single-cell level during transplant rejection and tolerance albeit with a common expression of the markers, CD4 and FOXP3.

Our current knowledge of Treg phenotypes is insufficient to define their cellular states correlated to transplant outcome. Recent advances in single-cell transcriptomics offer a new opportunity to discover additional subsets and cellular states of immune cells during development and disease pathogenesis at high resolution [48–50]. However, to date, there is no single-cell transcriptomic profiling data for Treg particularly in the transplantation setting. Here, we utilized the power of microfluidic scRNA-seq to establish a relatively large-scale cellular transcriptomic atlas of CD4^+^ T cells during transplantation by profiling ~ 13,000 cells including both conventional Th and regulator Treg from rejecting and tolerated grafts. We first analyzed data with ~ 1000 randomly selected cells of each group to minimize the impact of cell number biases between different groups. It is noteworthy that we also observed the same conclusion by analyzing all cells (~ 14,000 cells, Additional file 1: Figure S7). Our results offered unbiased genomic classification together with phenotypic and functional validation, leading us to revise the taxonomy of these cells in determining the transplant outcome.

Transcriptomically, we did pairwise comparisons. By comparing to Th, intragraft Treg expressed less genes that activated adaptive immunity such as B/T cell proliferation and NK cell chemotaxis during transplant tolerance. Likewise, they expressed more genes associated with negative regulation of conventional T cell function and less genes that activated adaptive immunity during transplant rejection. Since intragraft Treg still appeared to harbor immunomodulatory function, they were not bystanders during transplant rejection. So why might the grafts be rejected in the presence of Treg? We compared Treg derived from rejecting and tolerated grafts. Our data demonstrated cellular heterogeneity among Treg in the rejecting grafts: 48% Treg in C2 reflected a potential for chemotaxis of NK cells (*Ccl3*, *Ccl4*, *Ccl5*, *Xcl1*), monocytes (*Ccl3*, *Ccl4*, *Ccl9*, *Ccr2*, *Calca*, *Xcl1*), and neutrophils (*Ccl3*, *Ccl4*, *Ccl5*, *Ccl9*, *Itga1*, *Pde4b*, *Slc37a4*), and 37% Treg in C3 reflected a potential for protein ubiquitination and responses to interferons. In fact, the chemotactic genes *Ccl5* and *Itga1* were also overexpressed in splenic Treg of recipients that had rejecting grafts compared to that of the tolerated group. Furthermore, by comparing Th during rejection and tolerance, we might infer that Th negatively regulated the immune system and supported Treg function during tolerance.

Since scRNA-seq data revealed that 40% Treg of tolerated grafts were found in S-G2/M phages of the cell cycle, Treg proliferation was a possible major mechanism by which coreceptor and costimulation blockade mediated transplant tolerance. Indeed, we confirmed by immunostaining that > 80% FOXP3^+^ cells expressed Ki67 in the tolerated grafts compared to ~ 35% in the rejecting grafts. However, the signaling pathway driving any Treg proliferation during transplant tolerance is not clear. A previous report shows that the inhibitory checkpoint molecule PD-1 is vital in maintaining peripheral tolerance as PD-1 knockout mice spontaneously develop autoimmunity with markedly augmented proliferation of conventional T cells [51]. Since PD-L1 is found upregulated in many types of tumors, and PD-1 receptor is expressed by conventional T cells, it was hypothesized that tumors evaded immunosurveillance through the PD-L1/PD-1 pathway. Indeed, it is well characterized that signaling through PD-1 contributes to exhaustion and dysfunction of conventional T cells [31, 52], and anti-PD-1 mAb-mediated immunotherapy (e.g., Nivolumab) is currently used to treat human cancers [53]. In immune regulation, PD-1 expression on Treg is found inversely correlated to their proliferation during chronic liver inflammation [54], while in another study, PD-1 signaling promotes differentiation of CD4^+^ naïve [55] or Th1 [56] cells into induced Treg (iTreg) with suppressive function. Such conversion can operate with [57] or without [55] TGF-β. Nevertheless, the direct role of PD-1 in survival and/or function of Treg is less clear.

Our scRNA-seq data with subsequent validation by flow cytometry revealed that a significantly greater percentage of Treg expressed PD-1 during transplant tolerance than rejection. We found that blocking PD-1 signaling via the neutralizing anti-PD-1 antibody abolished coreceptor and costimulation blockade-induced transplant tolerance, resulting in rejection of hESC-derived tissues with significantly reduced proliferation of intragraft Treg. Therefore, our results suggested that PD-1 signaling could be one of the mechanisms by which antibody blockade mediated Treg proliferation. Nevertheless, it is difficult to examine the effect of PD-1 blockade on conventional T cells in the absence of Treg in the transplantation setting, as we showed that Treg were indispensable for antibody blockade-mediated immune tolerance to hESC grafts and their absence contributed to graft rejection. Given the differential expression of PD-1 on conventional T cells and Treg during transplant rejection and tolerance, there might in principle be some differential binding and therefore efficacy of anti-PD-1 antibody on conventional T cells and Treg during the tolerance induction process. Our results suggest a therapeutic potential of PD-1 agonists in promoting transplant tolerance to hESC-derived tissues given their effect in facilitating self-renewal of Treg. Indeed, a previous report demonstrated that overexpression of PD-L1 prevents transplant rejection of mouse islets [58]. Whether the same regime could prevent rejection of human islets awaits further investigations. Although the role of therapeutic anti-PD-1 antibody on Treg has yet to be studied in cancer patients [59], our results also give new insights into the unwanted side effect of PD-1 blockade that may limit self-renewal of Treg during cancer treatment. Since Treg suppress autoreactive T cells, PD-1 blockade may cause autoimmune attack as seen in cancer patients who received anti-PD-1 antibodies and developed T1D and other autoimmune diseases [30].

## Conclusions

Taken together, we have demonstrated that coreceptor and costimulation blockade induced transplant tolerance to hESC-derived pancreatic islets in NOD mouse recipients by promoting Treg proliferation via the PD-1 signaling pathway. These findings pave the way for clinical development of hESC-derived pancreatic tissues, combined with immunotherapies that expand intragraft Treg, as a potential treatment for alleviating autoimmunity in T1D. Our revised taxonomy of Treg during transplant rejection and tolerance might also enable more accurate monitoring of the outcome of solid organ transplantation.

## Additional file

Additional file 1: Single-cell transcriptomics reveal that PD-1 mediates immune tolerance by regulating proliferation of regulatory T cells, Supplementary Figures S1–7 and Tables S1–6. (DOCX 13159 kb)
