# Supplementary material for: Single-cell transcriptomics reveal that PD-1 mediates immune tolerance by regulating proliferation of regulatory T cells
Source: Genome Med. 2018 Sep 20;10:71. doi: 10.1186/s13073-018-0581-y (PMC6148788; doi:10.1186/s13073-018-0581-y)
Supplement: Supplementary file 1 — Single-cell transcriptomics reveal that PD-1 mediates immune tolerance by regulating proliferation of regulatory T cells, Supplementary Figures S1–7 and Tables S1–6. (DOCX 13159 kb) [file 13073_2018_581_MOESM1_ESM.docx]

**Figure S1 Coreceptor and costimulation blockade facilitates survival of hESC-EBs in NOD.*Foxp3*^hCD2^ mice.** (A) A representative image of hESC-derived teratoma formed 1 months post transplantation under the kidney capsule following treatment with anti-CD4, -CD8 and -CD40L mAbs (n=10). (B-D) Representative H&E images showing tissues of the (B) ectoderm, (C) endoderm and (D) mesoderm lineages. Arrows indicate the respective tissue structures. Scale bar = 100 uM.

**Figure S2 Genome-wide transcriptomic profiling of splenic CD4^+^** **Treg during transplant rejection and tolerance** **by bulk RNA-seq.** (A) Scatter plot showing correlation of the gene expression levels of splenic CD4^+^hCD2^+^ Treg respectively purified from the 3 mAb + αhCD2 mAb-treated rejecting (R-T_R_, n=3) and 3 mAb-treated tolerated (T-T_R_, n=3) grafts of NOD.*Foxp3*^hCD2^ post transplantation. (B) Heatmap showing the 43 differentially-expressed genes by comparing R-T_R_ and T-T_R_. GO functional annotations showing that the most significantly downregulated genes in T-T_R_ compared to R-T_R_ were associated in the following pathways: GO:0030593 neutrophil chemotaxis; GO:0045766 positive regulation of angiogenesis; and GO:0042102 positive regulation of T-cell proliferation.

**Figure S3 Random selection and filtering ~1000 cells per sample for detailed scRNAseq analysis.** (A) Distribution of total mRNAs expressed by CD4^+^ T-cells in both rejecting and tolerated grafts. Two vertical lines indicate the two standard deviations away from the mean. (B) Distribution of cells during filtering.

**
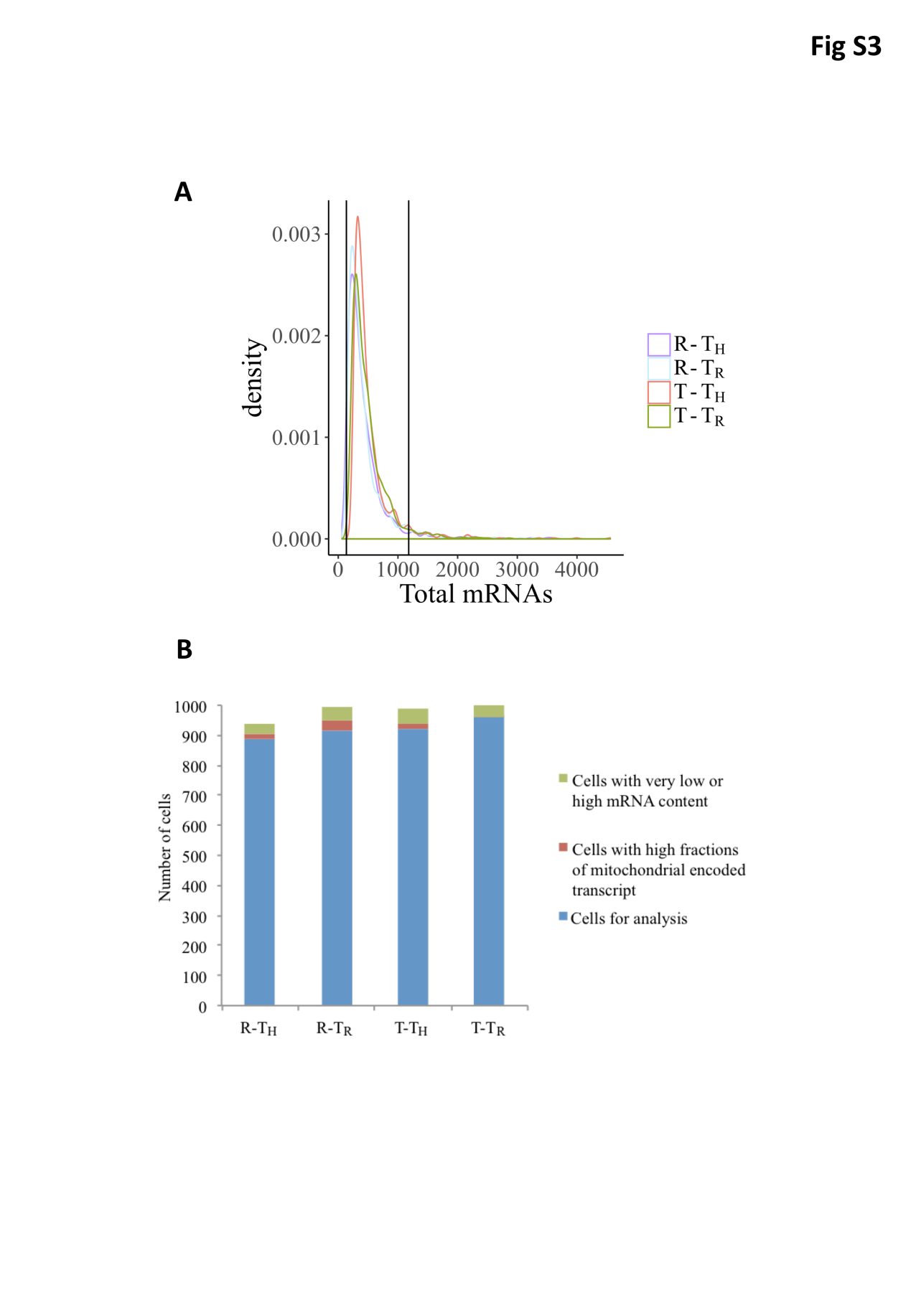
**

**Figure S4** **Identification of subset-specific signature genes expressed by CD4^+^ T-cells in both rejecting and tolerated grafts.** Jitter plots comparing the expression levels of various subset-specific genes of CD4^+^ (A) effector and (B) regulatory T-cells in CD4^+^hCD2^-^ Th of rejecting grafts (R-T_H_), CD4^+^hCD2^+^ Treg of rejecting grafts (R-T_R_), CD4^+^hCD2^-^ Th of tolerated grafts (T-T_H_) and CD4^+^hCD2^+^ Treg of tolerated grafts (T-T_R_).


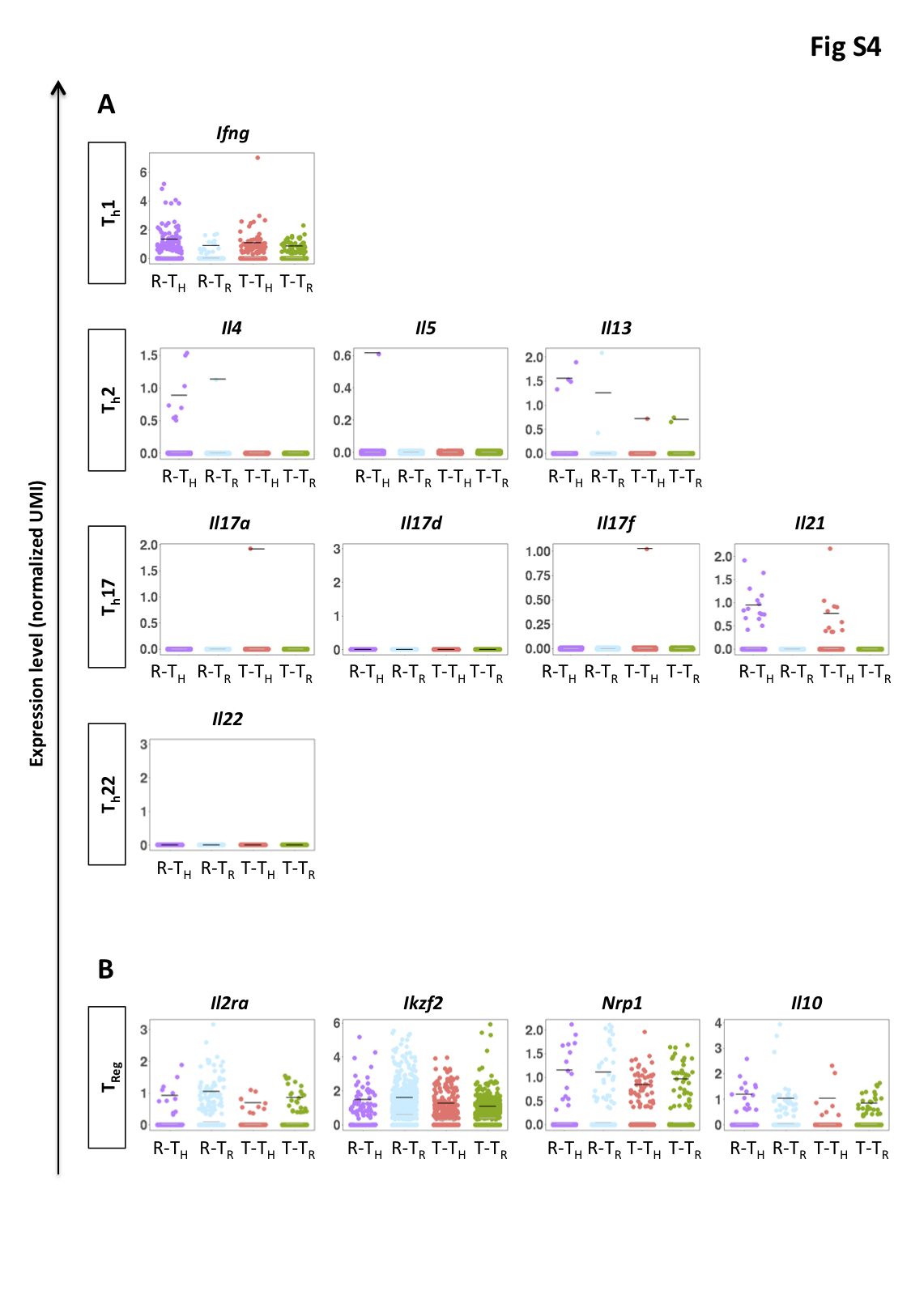


**Figure S5 Genome-wide transcriptomic profiling of intragraft CD4^+^** **Th and Treg during transplant rejection and tolerance at single-cell resolution.** (A, C, E) Biaxial scatter plots by *t*-SNE analysis showing single-cell transcriptomic clustering of CD4^+^hCD2^-^ (T_H_) or CD4^+^hCD2^+^ (T_R_) cells purified from rejecting (R) or tolerated (T) grafts. Cells were colored individually according to their initial expression of CD4 and hCD2 by FACS sorting. (B, D, F) Pathway analysis based on Gene Ontology functional annotations in terms of biological processes showing comparison of (B) T_H_ of tolerated to rejecting grafts; (D) T_R_ to T_H_ of rejecting grafts; or (F) T_R_ to T_H_ of tolerated grafts. Abbreviations: R-T_H_, T_H_ of rejecting grafts; R-T_R_, T_R_ of rejecting grafts; T-T_H_, T_H_ of tolerated grafts; and T-T_R_, T_R_ of tolerated grafts.


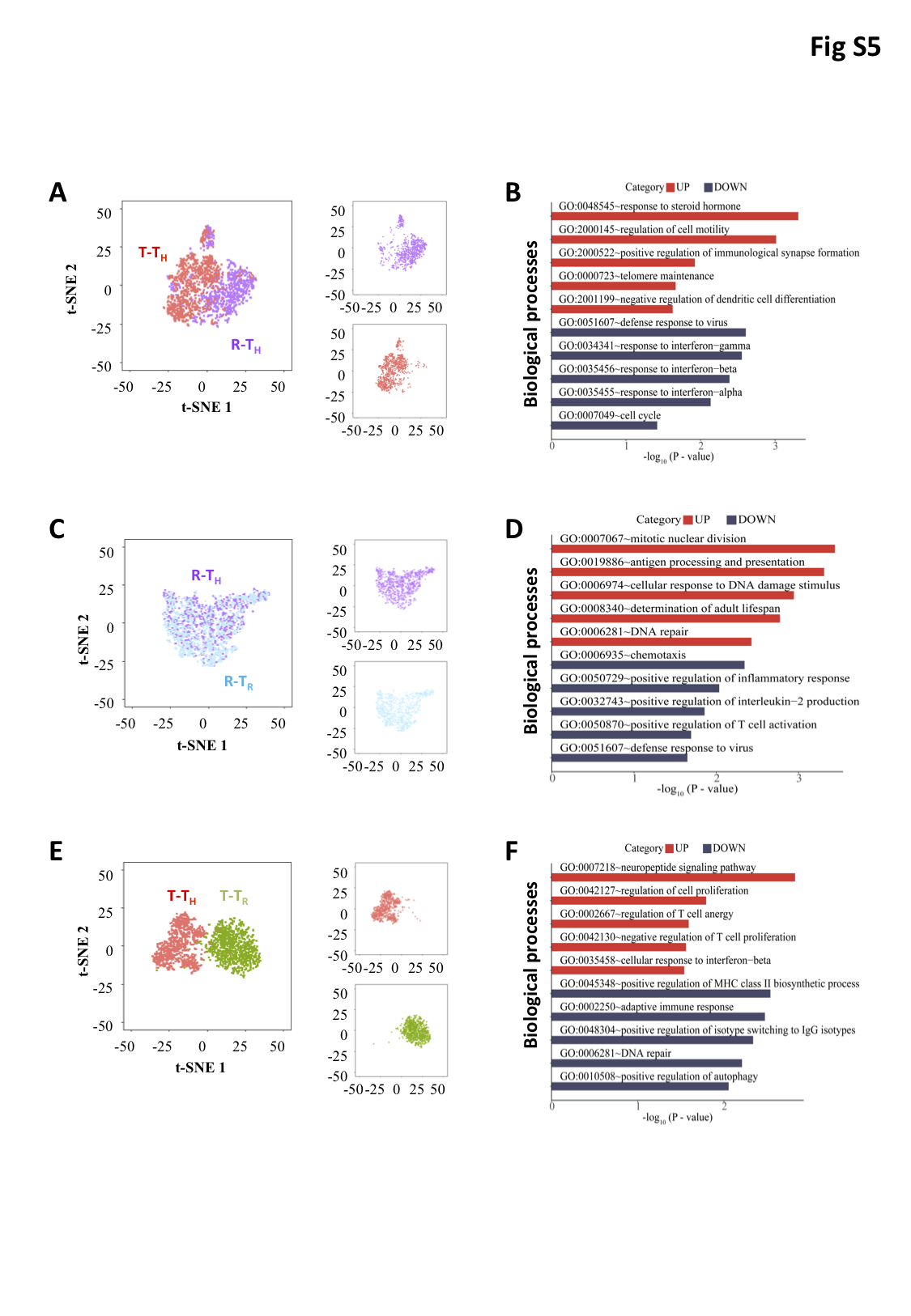


**Figure S6 Intragraft CD4^+^** **Treg are phenotypically and functionally distinct during transplant rejection and tolerance.** CD4^+^hCD2^+^ Treg were isolated from tolerated or rejecting grafts. Pathway analysis based on Gene Ontology functional annotations in terms of biological processes showing up- and down-regulated pathways in (A) C1 vs C2 and C3; (B) C2 vs C1 and C3; or (C) C3 vs C1 and C2. Abbreviations: C1, cluster 1; C2, cluster 2; and C3, cluster 3


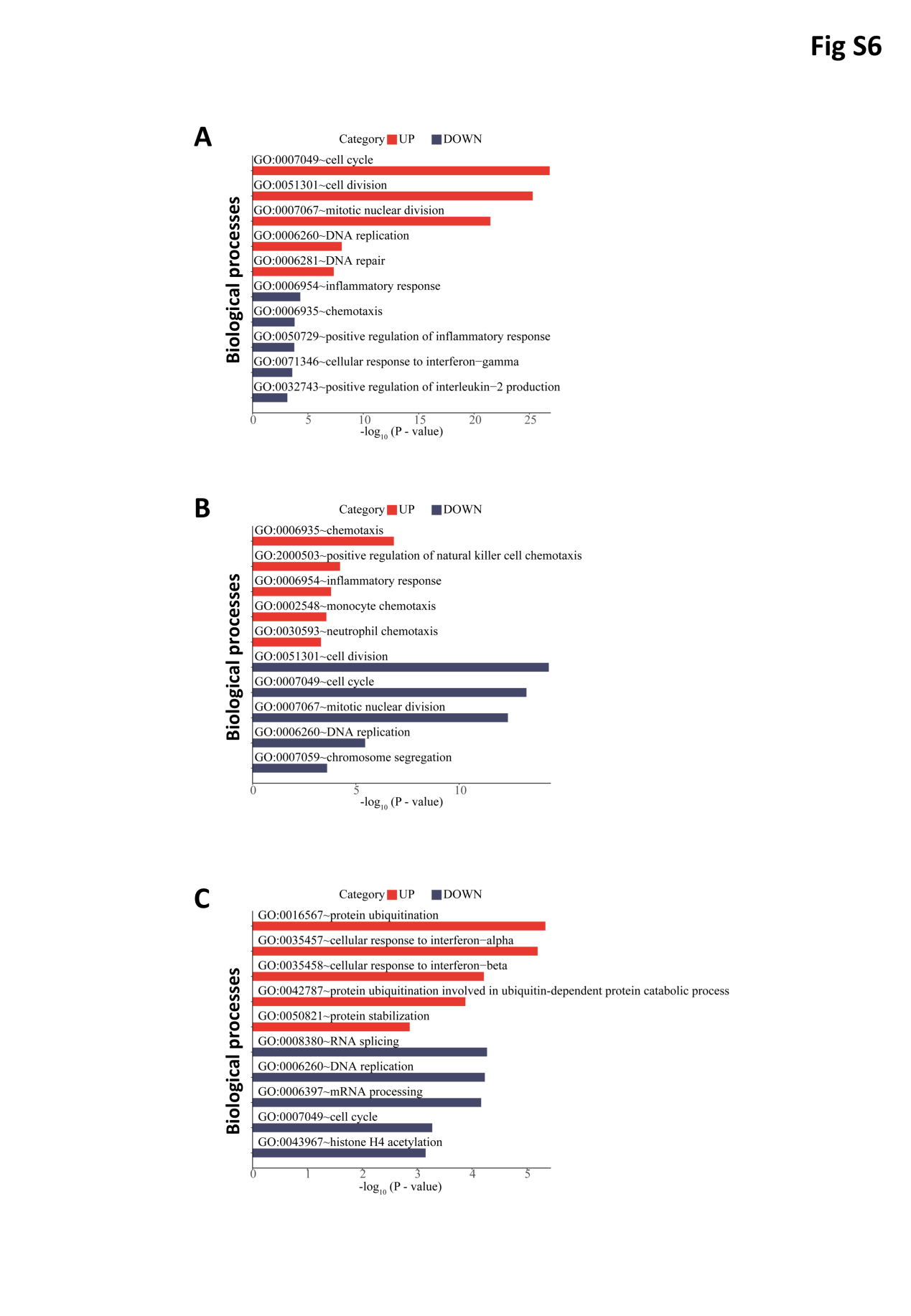


**Figure S7 Detailed scRNAseq analysis of all cells shows the same conclusion compared to that of randomly selected ~1,000 cells per sample.** (A, in relation to Fig 3A) Biaxial scatter plots by *t*-SNE analysis showing single-cell transcriptomic clustering of 858 CD4^+^hCD2^-^ (R-T_H_) and 954 CD4^+^hCD2^+^ (R-T_R_) cells respectively purified from rejecting grafts; and 3,654 CD4^+^hCD2^-^ (T-T_H_) and 7,498 CD4^+^hCD2^+^ (T-T_R_) cells respectively purified from tolerated grafts. Cells were colored individually according to their initial expression of CD4 and hCD2 during FACS sorting. (B-D) Pathway analysis based on Gene Ontology functional annotations in terms of biological processes showing comparison of (B, in relation to Figure S5B) T_H_ of tolerated to rejecting grafts; (C, in relation to Figure S5D) T_R_ to T_H_ of rejecting grafts; or (D, in relation to Figure S5F) T_R_ to T_H_ of tolerated grafts. (E, in relation to Figure 4B) Jitter plot comparing expression levels of *Pdcd1* expressed by R-T_H_, R-T_R_, T-T_H_ and T-T_R_. The fold change (FC) of T-T_R_ over R-T_R_, and the p-value (P) by sSeq method are provided. Grey and black bars indicate the average expression level among all and expressed cells, respectively.

**
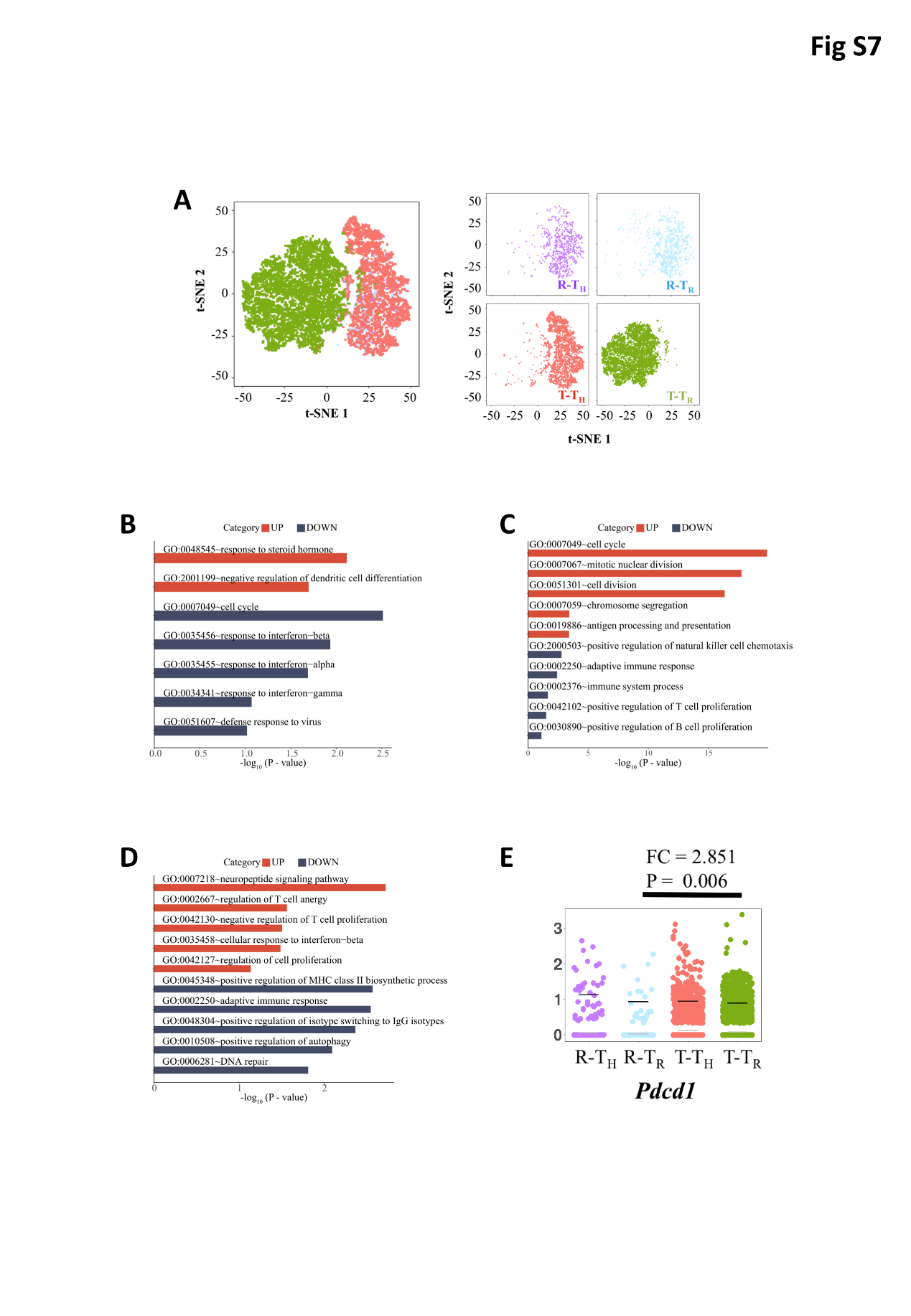
**

**Supplemental Tables**

**Table S1** **GO functional annotations showing a list of genes expressed by Th cells purified from tolerated (T-T_H_) compared to that of rejecting (R-T_H_) hESC-derived grafts with the top 5 most significantly upregulated and downregulated pathways, respectively, in terms of biological processes as determined by scRNA-seq.**

| **Upregulated Pathway** | **p-value** | **Molecules** |
| --- | --- | --- |
| GO:0048545~response to steroid hormone | 4.87E-04 | Spp1, Car2, Bcl2, Abca2 |
| GO:2000145~regulation of cell motility | 9.67E-04 | Cd81, Arhgap18, Igsf8 |
| GO:2000522~positive regulation of immunological synapse formation | 0.011910235 | Clec2i, Ccr7 |
| GO:0000723~telomere maintenance | 0.021604957 | Prkdc, Dclre1b, Pot1a |
| GO:2001199~negative regulation of dendritic cell differentiation | 0.023679915 | Tmem176a, Tmem176b |
| **Downregulated Pathway** | **p-value** | **Molecules** |
| GO:0051607~defense response to virus | 0.002464859 | Bnip3, Slfn8, Stat2, Ifitm1, Ifit3b, Ifitm3, Cd86, Ifitm2 |
| GO:0034341~response to interferon-gamma | 0.002792642 | Ifitm1, Ifitm3, Cd86, Ifitm2 |
| GO:0035456~response to interferon-beta | 0.004082932 | Ifitm1, Ifitm3, Ifitm2 |
| GO:0035455~response to interferon-alpha | 0.00732537 | Ifitm1, Ifitm3, Ifitm2 |
| GO:0007049~cell cycle | 0.038165134 | Dscc1, Plk3, Ube2s, Cdca2, Dlgap5, Kif2c, Nuf2, Haus5, Brca1, Prkcd, Pard6a, Cdkn1a, Terf2 |

**Table S2** **GO functional annotations showing a list of genes expressed by Treg (R-T_R_) compared to Th (R-T_H_) cells of rejecting hESC-derived grafts with the top 5 most significantly upregulated and downregulated pathways, respectively, in terms of biological processes as determined by scRNA-seq.**

| **Upregulated Pathway** | **p-value** | **Molecules** |
| --- | --- | --- |
| GO:0007218~neuropeptide signaling pathway | 0.001492157 | Gcat, Penk, Ltb4r1, Cysltr1, Calca |
| GO:0042127~regulation of cell proliferation | 0.016081284 | Bid, Bmpr2, Cd81, Prg4, Tec, Mafg |
| GO:0002667~regulation of T cell anergy | 0.025634074 | Phlpp1, Foxp3 |
| GO:0042130~negative regulation of T cell proliferation | 0.027592255 | Sdc4, Il2ra, Foxp3 |
| GO:0035458~cellular response to interferon-beta | 0.028886582 | Gbp2, F830016B08Rik, Gbp2b |
| **Downregulated Pathway** | **p-value** | **Molecules** |
| GO:0045348~positive regulation of MHC class II biosynthetic process | 0.0028893 | Cd40lg, Il4, Ifng |
| GO:0002250~adaptive immune response | 0.003344768 | Trat1, Cracr2a, Il2, Ifng, Sema4a, Cd8b1, Slamf7 |
| GO:0048304~positive regulation of isotype switching to IgG isotypes | 0.004580382 | Il2, Il4, Ifng |
| GO:0006281~DNA repair | 0.006169181 | Fancg, Mre11a, Neil3, Uvrag, Ino80, Usp45, Cdk2, Nudt1, Brca1, Prmt6 |
| GO:0010508~positive regulation of autophagy | 0.008822648 | Tbc1d5, Uvrag, Ifng, Nprl2 |

**Table S3 GO functionally annotations showing a list of genes expressed by Treg (T-T_R_) compared to Th (T-T_H_) cells of tolerated hESC-derived grafts with the top 5 most significantly upregulated and downregulated pathways, respectively, in terms of biological processes as determined by scRNA-seq.**

| **Upregulated Pathway** | **p-value** | **Molecules** |
| --- | --- | --- |
| GO:0007049~cell cycle | 1.76E-13 | Cep55, Fbxo5, Dlgap5, Cdca2, Kif2c, Cdk1, Haus5, Spc24, Spc25, Pim1, Ube2c, Cdc20, Terf2, Ndc80, Aurka, Dscc1, Ska1, Kif11, Sass6, Nuf2, Esco2, Sgol1, Mcm5, Cdc45, Ncaph, Pard6a, Melk, Casc5, Pkmyt1, Apitd1 |
| GO:0051301~cell division | 2.16E-11 | Cep55, Aurka, Ska1, Fbxo5, Kif11, Cdca2, Kif2c, Cdk1, Nuf2, Haus5, Spc24, Sgol1, Spc25, Mcm5, Cdc45, Ncaph, Pard6a, Ube2c, Cdc20, Casc5, Ndc80, Apitd1 |
| GO:0007067~mitotic nuclear division | 5.97E-11 | Cep55, Aurka, Ska1, Fbxo5, Kif11, Cdca2, Kif2c, Cdk1, Nuf2, Haus5, Spc24, Sgol1, Spc25, Ncaph, Ube2c, Cdc20, Casc5, Ndc80, Apitd1 |
| GO:0007059~chromosome segregation | 1.33E-06 | Sgol1, Spc25, Ska1, Kif11, Cdca2, Kif2c, Casc5, Ndc80, Esco2 |
| GO:0019886~antigen processing and presentation of exogenous peptide antigen via MHC class II | 5.79E-06 | H2-Aa, Cd74, H2-Ab1, Ifi30, H2-Eb1 |
| **Downregulated Pathway** | **p-value** | **Molecules** |
| GO:0030890~positive regulation of B cell proliferation | 1.739130435 | Cd40lg, Nckap1l, Tnfrsf13c, Bcl2, Bmi1, Tnfsf13b |
| GO:0002250~adaptive immune response | 2.608695652 | Trat1, Cd7, Tfe3, Klrk1, Tnfrsf13c, Jak2, Sema4a, Tsc1, Eomes |
| GO:2000503~positive regulation of natural killer cell chemotaxis | 0.869565217 | Ccl5, Ccl3, Xcl1 |
| GO:0042102~positive regulation of T cell proliferation | 1.739130435 | Cd40lg, Nckap1l, Ccl5, Tnfrsf13c, Tnfsf13b, Cd274 |
| GO:0002376~immune system process | 4.057971014 | Tfe3, Jak2, Tnfrsf13c, Tbk1, Sema4a, Eomes, Otulin, Trat1, Mavs, Pik3cg, Cd7, Klrk1, Gbp5, Gbp2b |

**Table S4** **GO functionally annotations showing a list of genes expressed by C1 of Treg purified from hESC-derived grafts with the top 5 most significantly upregulated and downregulated pathways, respectively, in terms of biological processes as determined by** ***t*-SNE analysis.**

| **Upregulated Pathway** | **p-value** | **Molecules** |
| --- | --- | --- |
| GO:0007049~cell cycle | 1.45E-27 | Cdkn3, Uhrf1, Fbxo5, Cdc14a, Spc25, Nup43, Cks2, Ccnb2, Gmnn, Cdc20, Rad50, Cenpw, Cdca8, Mis18bp1, Rbl1, Cdk4, Timeless, Pelo, Mcmbp, Mki67, Ccnb1, Nusap1, Mad2l1, Apitd1, Cdkn2a, Cdca3, Ska2, Cdca2, Prc1, Cdk1, Pttg1, Spc24, Nup37, Knstrn, Cdc25b, Mcm7, Ube2c, Ccne2, Smc2, Ncapd2, Cks1b, Mcm2, Birc6, Racgap1, Mis12, Aurkb, Birc5, Cenpe, Ppm1g, Esco2, Sgol1, Hells, Mcm5 |
| GO:0051301~cell division | 4.95E-26 | Ska2, Cdca3, Fbxo5, Cdca2, Prc1, Cdk1, Pttg1, Spc24, Nup37, Cdc14a, Spc25, Nup43, Cks2, Ccnb2, Knstrn, Cdc25b, Ube2c, Cdc20, Ccne2, Smc2, Ncapd2, Cks1b, Cenpw, Cdca8, Mis18bp1, Racgap1, Birc6, Cdk4, Mis12, Aurkb, Birc5, Cenpe, Pelo, Timeless, Mcmbp, Sgol1, Hells, Mcm5, Ccnb1, Nusap1, Mad2l1, Apitd1 |
| GO:0007067~mitotic nuclear division | 3.33E-22 | Cdca3, Ska2, Fbxo5, Cdca2, Cdk1, Pttg1, Spc24, Nup37, Cenph, Spc25, Nup43, Ccnb2, Knstrn, Cdc25b, Ube2c, Cdc20, Smc2, Ncapd2, Cenpw, Cdca8, Mis18bp1, Birc6, Mis12, Aurkb, Birc5, Cenpe, Timeless, Mcmbp, Sgol1, Hells, Ccnb1, Nusap1, Mad2l1, Apitd1 |
| GO:0006260~DNA replication | 5.223880597 | Ino80e, Pold2, Mcm2, 2810417H13Rik, Pcna, Fen1, Mcmbp, Gins2, Mcm7, Mcm5, Rfc3, Pola2, Rfc5, Rrm2 |
| GO:0006281~DNA repair | 7.462686567 | Rad50, Ino80e, Xrcc6, Uhrf1, Uvrag, Dclre1c, 2810417H13Rik, Stub1, Pttg1, Dmap1, Pcna, Fen1, 3110062M04Rik, Faap24, Fancg, Mre11a, Ung, Apex1, Rdm1, Apitd1 |
| **Downregulated Pathway** | **p-value** | **Molecules** |
| GO:0006954~inflammatory response | 4.87E-05 | Ccl4, Ccl9, Tnfrsf1a, Calca, Pik3cg, Ccr3, Ecm1, Csf1, Ccr2, Plaa, Ccl3, Cysltr1, Tnfrsf14, Ccr5, Gbp5, Prkcz |
| GO:0006935~chemotaxis | 1.59E-04 | Pik3cg, Ccr3, Ccl4, Ccl9, Ccr2, Cysltr1, Ccl3, Ccr5, Ccr9 |
| GO:0050729~positive regulation of inflammatory response | 1.69E-04 | Il1rl1, Ccl4, Ccl9, Ccr2, Tnfrsf1a, Ccl3, Ccr5 |
| GO:0071346~cellular response to interferon-gamma | 2.58E-04 | Ccl4, Gbp3, Ccl9, Ccl3, Gbp8, Gbp5, Gbp2b |
| GO:0032743~positive regulation of interleukin-2 production | 7.18E-04 | Map3k7, Ccr2, Malt1, Pde4b |

**Table S5** **GO functionally annotations showing a list of genes expressed by C2 of Treg purified from hESC-derived grafts with the top 5 most significantly upregulated and downregulated pathways, respectively, in terms of biological processes as determined by *t*-SNE analysis.**

| **Upregulated Pathway** | **p-value** | **Molecules** |
| --- | --- | --- |
| GO:0006935~chemotaxis | 1.46E-07 | Pik3cg, Ccr3, Ccl4, Ccl5, Ccl9, Ccr2, Cysltr1, Ccl3, Pip5k1c, Ccr5, Cx3cr1, Ccr9, Xcl1 |
| GO:2000503~positive regulation of natural killer cell chemotaxis | 5.82E-05 | Ccl4, Ccl5, Ccl3, Xcl1 |
| GO:0006954~inflammatory response | 1.58E-04 | Ccl4, Ccl9, Calca, Pik3cg, Ccr3, Ecm1, Ccl5, Ccr2, Ccl3, Cysltr1, Tnfrsf14, Havcr2, Il10, Ccr5, Xcl1, Prkcz |
| GO:0002548~monocyte chemotaxis | 2.67E-04 | Ccl4, Ccl9, Ccr2, Ccl3, Xcl1, Calca |
| GO:0030593~neutrophil chemotaxis | 4.88E-04 | Ccl4, Slc37a4, Ccl5, Ccl9, Ccl3, Pde4b, Itga1 |
| **Downregulated Pathway** | **p-value** | **Molecules** |
| GO:0051301~cell division | 4.62E-15 | Cdca3, Ube2s, Fbxo5, Cdk1, Spc24, Ccnb2, Cdc25b, Ube2c, Reep4, Cdc20, Ccne2, Smc2, Cks1b, Cdca8, Tacc1, Racgap1, Cdk4, Birc5, Cenpe, Mcmbp, Sgol1, Hells, Ccnb1, Nusap1, Mad2l1, Apitd1, Ruvbl1 |
| GO:0007049~cell cycle | 5.57E-14 | Cdkn2a, Ube2s, Cdca3, Fbxo5, Cdk1, Spc24, Ccnb2, Cdc25b, Gmnn, Reep4, Ube2c, Gadd45gip1, Cdc20, Ccne2, Smc2, Cks1b, Mcm2, Cdca8, Tacc1, Racgap1, Cdk4, Birc5, Cenpe, Esco2, Mcmbp, Sgol1, Hells, Ccnb1, Nusap1, Mad2l1, Apitd1, Ruvbl1 |
| GO:0007067~mitotic nuclear division | 4.41E-13 | Smc2, Cdca3, Cdca8, Fbxo5, Cdk1, Spc24, Cenph, Birc5, Cenpe, Mcmbp, Sgol1, Hells, Ccnb2, Cdc25b, Ccnb1, Reep4, Ube2c, Cdc20, Nusap1, Mad2l1, Ruvbl1, Apitd1 |
| GO:0006260~DNA replication | 3.56E-06 | Mcmbp, Ino80e, Pold3, Mcm2, Gins2, Rfc3, 2810417H13Rik, Rfc5, Fen1, Rrm2 |
| GO:0007059~chromosome segregation | 2.47E-04 | Sgol1, Ppp1r7, Birc5, Cenph, Cenpe, Cenpf, Esco2 |

**Table S6 GO functionally annotations showing a list of genes expressed by C3 of Treg purified from hESC-derived grafts with the top 5 most significantly upregulated and downregulated pathways, respectively, in terms of biological processes as determined by *t*-SNE analysis.**

| **Upregulated Pathway** | **p-value** | **Molecules** |
| --- | --- | --- |
| GO:0016567~protein ubiquitination | 4.62E-06 | Tnfaip3, Neurl3, Rnf123, Rnf19b, Syvn1, Dcaf10, Dcaf5, Bach1, Rnf38, Ltn1, Trim21, Trim41, Dzip3, Siah1a, Trim25, Ubr7, Ankib1, March7, Kbtbd11, Fbxw7, Tnfaip1, Traf3, Asb13, Klhl15, Fbxo33, Fbxl5 |
| GO:0035457~cellular response to interferon-alpha | 6.40E-06 | Ifit3b, Ifi204, Axl, Ifit3, Ifit2, Ifit1 |
| GO:0035458~cellular response to interferon-beta | 6.10E-05 | Gbp6, Ifi204, Tgtp1, F830016B08Rik, Ifit3, Ifit1, Iigp1, Gbp2b |
| GO:0042787~protein ubiquitination involved in ubiquitin-dependent protein catabolic process | 1.32E-04 | Siah1a, Dzip3, Rnf11, Syvn1, Trim25, Ankib1, Asb2, Klhl15, Gm15800, Arel1, Hectd2, Rmnd5a, Rnf19b |
| GO:0050821~protein stabilization | 0.001370755 | Syvn1, Cog3, Per3, Stk3, Tsc1, Sav1, Tesc, Fbxw7, Rassf2, Gnaq, Hps4 |
| **Downregulated Pathway** | **p-value** | **Molecules** |
| GO:0008380~RNA splicing | 5.35E-05 | Zranb2, Rbmxl1, Dcps, Ncbp2, Wbp4, Lsm3, Ddx39b, Sf1, Ppie |
| GO:0006260~DNA replication | 5.86E-05 | Fam111a, Lig1, Rfc2, Mcm5, Dtl, 2810417H13Rik, Pcna |
| GO:0006397~mRNA processing | 6.84E-05 | Zranb2, Rbmxl1, Dcps, Ncbp2, Wbp4, Lsm3, Ddx39b, Sf1, Cpsf2, Ppie |
| GO:0007049~cell cycle | 5.32E-04 | Lig1, Smc2, Anapc10, Cdc25b, Gmnn, Mcm5, Babam1, Usp16, Anapc5, Nusap1, Smarcb1, Ppm1g |
| GO:0043967~histone H4 acetylation | 7.04E-04 | Morf4l2, Brd8, Yeats4, Hat1 |
